# Predictors of renal flares in systemic lupus erythematosus: a post-hoc analysis of four phase III clinical trials of belimumab

**DOI:** 10.1093/rheumatology/keae023

**Published:** 2024-01-12

**Authors:** Sandra Jägerback, Alvaro Gomez, Ioannis Parodis

**Affiliations:** Division of Rheumatology, Department of Medicine Solna, Karolinska Institutet and Karolinska University Hospital, Stockholm, Sweden; Division of Rheumatology, Department of Medicine, Danderyd University Hospital, Danderyd, Sweden; Division of Rheumatology, Department of Medicine Solna, Karolinska Institutet and Karolinska University Hospital, Stockholm, Sweden; Division of Rheumatology, Department of Medicine Solna, Karolinska Institutet and Karolinska University Hospital, Stockholm, Sweden; Department of Rheumatology, Faculty of Medicine and Health, Örebro University, Örebro, Sweden

**Keywords:** systemic lupus erythematosus, biomarkers, renal flares, belimumab, biologics

## Abstract

**Objective:**

The objective of this study was to identify predictors of renal flares in patients with SLE treated for active extra-renal disease.

**Methods:**

Data from four clinical trials of belimumab in SLE (BLISS-52, NCT00424476; BLISS-76, NCT00410384; BLISS-NEA, NCT01345253; BLISS-SC, NCT01484496) were used. Patients were assigned to belimumab or placebo on top of standard therapy. We investigated the performance of predictors of renal flares through weeks 52–76 using proportional hazards regression analysis.

**Results:**

Of 3225 participants, 192 developed at least one renal flare during follow-up, with the first occurring after a median time of 197 days. Current/former renal involvement [hazards ratio (HR): 15.4; 95% CI: 8.3–28.2; *P* < 0.001], low serum albumin levels (HR 0.9; 95% CI: 0.8–0.9; *P* < 0.001), proteinuria (HR: 1.6; 95% CI: 1.5–1.7; *P* < 0.001), and low C3 levels (HR: 2.9; 95% CI: 2.1–4.1; *P* < 0.001) at baseline appeared robust determinants of impending renal flares. Anti-dsDNA positivity yielded an increased hazard for renal flares (HR: 2.1; 95% CI: 1.4–3.2; *P* < 0.001), which attenuated after adjustments. Anti-Sm positivity was associated with renal flares in the placebo (HR: 3.7; 95% CI: 2.0–6.9; *P* < 0.001) but not in the belimumab subgroup, whereas anti-ribosomal P positivity was associated with renal flares in the belimumab subgroup only (HR: 2.8; 95% CI: 1.5–5.0; *P* = 0.001).

**Conclusion:**

A history of renal involvement, high baseline proteinuria, hypoalbuminaemia, and C3 consumption were robust determinants of impending renal flares. In addition to anti-dsDNA, anti-Sm and anti-ribosomal P protein antibody positivity may have value in surveillance of renal SLE.

Rheumatology key messages

- Nephritis history and high proteinuria predicted renal flares in SLE patients treated for extra-renal disease.
- C3 consumption and hypoalbuminaemia were determinants of impending renal flare in SLE patients.
- In addition to anti-dsDNA, anti-Sm and anti-ribosomal P protein antibodies may prove useful markers of renal SLE.

## Introduction

SLE is a chronic, multisystem autoimmune disease that is associated with substantial morbidity, poor health-related quality of life (HRQoL), and premature death [1]. Renal involvement due to SLE, termed LN, occurs in 30–60% of patients [2]. Renal flares lead to severe nephron loss, resulting in worsening of kidney function and shortening of the kidney life-span, which constitutes a major determinant of poor long-term prognosis [3–5]. Both glomerular and tubulointerstitial lesions, as well as intrarenal vascular pathology, have been shown to contribute to the loss of renal function [2, 6, 7].

SLE is characterized by an abnormal B lymphocyte function and autoantibody production [8]. Autoantibodies may be directly involved in the pathogenesis of SLE, initiating immune injury by triggering a cellular and cytokine response, or through immune complex formation and deposition in organs or tissues [8, 9]. B cell activating factor belonging to the TNF family (BAFF; also known as B lymphocyte stimulator, BLyS) is a B cell differentiation and survival factor, which, among other functions, supports autoreactive B cells and inhibits their deletion [10]. Belimumab is a human monoclonal antibody that targets BAFF and has been approved as an add-on therapy for patients with active, autoantibody-positive SLE, and since recently for the treatment of active LN [11].

Renal flares can develop despite immunosuppressive therapy, and the circumstances that lead up to a renal flare are largely unclear. Low levels of complement protein 3 (C3) and C4, high levels of anti- dsDNA antibodies, and increasing levels of proteinuria are features that are coupled with the development of renal flares [12]. Surveillance of renal SLE consists primarily of monitoring serum creatinine, proteinuria, haematuria, abnormalities in the urinary sediment, and changes in serological markers such as anti-dsDNA antibodies and complement levels [12–14]. Despite improved management with less toxic and more potent pharmaceuticals, LN still leads to end-stage kidney disease (ESKD) in 5–20% of the afflicted patients [2]. Therefore, identification of patient profiles with susceptibility to developing renal flares despite immunosuppressive therapy is needed towards individualizing pharmacotherapy, hence preventing renal flare development and subsequent renal function loss.

The aim of the present study was to identify factors that are associated with the development of renal flares in patients with SLE commencing treatment for active extra-renal disease.

## Patients and methods

### Study design and population

For the purpose of this post-hoc analysis, we used pooled data from four phase III randomized controlled trials (RCTs) of belimumab in patients with SLE, i.e. BLISS-52 (NCT00424476; *N* = 865) [15], BLISS-76 (NCT00410384; *N* = 819) [16], BLISS Northeast Asia (NEA; NCT01345253; *N* = 705) [17], and BLISS-SC (NCT01484496; *N* = 836) [18]. Data were made available through the Clinical Study Data Request (CSDR) consortium.

In these trials, patients with moderate to severe SLE activity were deemed eligible for participation, while active severe LN was an exclusion criterion. Participants were randomly assigned to i.v. belimumab 1 mg/kg or 10 mg/kg at baseline, week 2, week 4, and thereafter every fourth week (in BLISS-52, BLISS-76, and BLISS-NEA), s.c. belimumab 200 mg weekly (in BLISS-SC), or placebo.

To qualify for recruitment in the BLISS studies, patients were required to have an SLE diagnosis according to the 1997 ACR classification criteria [19], and an Estrogen in Lupus Erythematosus National Assessment version of the SLEDAI (SELENA-SLEDAI) [20] score of ≥6 in BLISS-52 and BLISS-76 and ≥8 in BLISS-SC and BLISS-NEA. Patients also had to have an ANA titre of ≥1:80 and/or anti-dsDNA antibody level of ≥30 IU/ml. Furthermore, patients needed to have been on stable non-biologic standard therapy (ST) for at least 30 days prior to initiating treatment with belimumab or placebo; this included glucocorticoids, antimalarial agents, and/or standard immunosuppressants. The similar study design and data collection across these four RCTs allowed us to combine data from all trials.

The study was conducted in compliance with the ethical principles of the Declaration of Helsinki. Written informed consent was obtained from all study participants prior to enrolment in the four different BLISS studies. The current investigation was approved by the Swedish Ethical Review Authority (reference: 2019–05498).

### Clinical chemistry and traditional serological markers

We evaluated serological markers [albumin, C3, C4, BAFF, anti-dsDNA, anti-Smith (anti-Sm), anti-ribosomal P protein, and aCL antibodies) as well as proteinuria as potential predictors of renal flares. Measurements were performed within the frame of the BLISS programmes.

Levels of C3, C4, BAFF, and autoantibodies were determined using ELISA. The cut-off for low C3 levels was set to <90 mg/dl in all four trials, and for C4 the cut-offs were set to <16 mg/dl in BLISS-52 and BLISS-76, and to <10 mg/dl in BLISS-SC and BLISS-NEA. The cut-off for positivity was set to ≥30 IU/ml for anti-dsDNA, ≥15 U/ml for anti-Sm, and >25 EU/ml for anti-ribosomal P protein in all trials. For aCL antibodies, cut-offs for positivity were as follows: IgG aCL: 10 GPL U/ml in BLISS-52 and BLISS-76, and ≥14 GPL U/ml in BLISS-SC and BLISS-NEA; IgM aCL: ≥10 MPL U/ml in BLISS-52 and BLISS-76, and ≥12 MPL U/ml in BLISS-SC and BLISS-NEA; IgA aCL: ≥15 APL U/ml in BLISS-52 and BLISS-76, and ≥11 APL U/ml in BLISS-SC and BLISS-NEA [15–18].

### Outcome and clinical assessments

The primary end point of the BLISS trials was attainment of Systemic Lupus Erythematosus Responder Index 4 (SRI-4) [21] at week 52, i.e. reduction of ≥4 points in SELENA-SLEDAI score, no new BILAG [22] A organ domain score and no more than one new B organ domain score, and no worsening in the SELENA-SLEDAI Physician’s Global Assessment (PGA) score of >10% compared with baseline.

The outcome of the present investigation was development of renal flares, defined according to the analysis plans within the BLISS programmes as (i) a reproducible increase in proteinuria to >1 g/day if the baseline value was <0.2 g/day, >2 g/day if the baseline value was 0.2–1.0 g/day, or >2 times the baseline value if the baseline value was >1 g/day, (ii) increase in serum creatinine of ≥20% or 0.3 mg/dl, accompanied by proteinuria, haematuria or red blood cell (RBC) casts, or (iii) new haematuria of glomerular origin, accompanied by proteinuria or RBC casts. Proteinuria was estimated by 24-h urine protein excretion, or the urine protein to creatinine ratio (UPCR).

### Statistical analysis

Descriptive statistics are presented as means (S.D.), or medians (interquartile range, IQR) for continuous variables, while frequencies (percentage) are reported for categorical variables.

Analysis of baseline data from the pooled population was performed for comparisons between patients who developed at least one renal flare and patients who did not. For comparisons of continuous variables, the Mann–Whitney *U* test was employed, whereas comparisons between binomial variables were performed using the chi-squared (*χ*^2^) or Fisher’s exact test. Proportional hazards (Cox) regression models were employed to investigate demographics, baseline clinical parameters, and traditional laboratory markers for their potential association with renal flare development. Multivariable models adjusting for age, sex, ethnicity, BMI, organ damage, baseline extra-renal disease activity (SLEDAI-2K score excluding renal and immunological descriptors), current or former renal SLE activity (renal BILAG A–D), baseline use of glucocorticoids, antimalarials, and immunosuppressants, and use of belimumab. We also performed subgroup analyses after stratification into belimumab and placebo recipients, as well as after stratification into patients with current or former renal involvement (renal BILAG A–D) and patients with no history of renal SLE until baseline (renal BILAG E). To further assess the importance of the different variables being assessed as predictors, we performed a random forest variable importance analysis using the randomForestSRC package.

*P* values below 0.05 were deemed statistically significant. All analyses were performed using the R version 4.01 software (R Foundation for Statistical Computing, Vienna, Austria). The GraphPad Prism software version 9 (La Jolla, California, USA) was used for creating forest plots, and the R version 4.01 software for plotting survival curves.

## Results

### Patient characteristics

Baseline characteristics for the pooled study population are detailed in Table 1. The mean age of the patients was 36.7 years, 94% were women, and 54.6% had current or former renal involvement at baseline (renal BILAG A–D). A total of 192 individuals developed at least one renal flare, with the first occurring after a median follow-up time of 197 (IQR: 85–330) days from baseline. Patients who developed at least one renal flare during follow-up were younger than patients who did not develop renal flares (32.1 *vs* 37.0 years; *P* < 0.001). Furthermore, higher proportions of patients of Asian origin (71.9% *vs* 36.4%; *P* < 0.001), and lower proportions of White/Caucasian patients (15.1% *vs* 41.9%; *P* < 0.001) and Indigenous American patients (8.3% *vs* 14.3%; *P* = 0.021) were seen among patients who developed renal flares compared with the non-flaring patient population.

**Table 1.** Patient characteristics and comparisons between patients who developed renal flares and patients who did not

	All patients	Renal flare	No renal flare	*P* value
	*N* = 3225	*N* = 192	*N* = 3033	
**Patient characteristics**				
Age at baseline (years)	36.7 ± 11.6	32.1 ± 10.6	37.0 ± 11.6	**<0.001**
BMI; kg/m²	25 ± 5.9	25.1 ± 5.9	23.5 ± 4.7	**<0.001**
Female sex	3030 (94%)	175 (91.1%)	2855 (94.1%)	0.127
Ancestry				
Asian	1242 (38.5%)	138 (71.9%)	1104 (36.4%)	**<0.001**
Black/African American	234 (7.3%)	9 (54.7%)	225 (7.4%)	0.157
Indigenous American^a^	449 (13.9%)	16 (8.3%)	433 (14.3%)	**0.021**
White/Caucasian	1300 (40.3%)	29 (15.1%)	1271 (41.9%)	**<0.001**
**Clinical data**				
SLE duration at baseline (years)	6.4 ± 6.2	6.1 ± 5.5	6.4 ± 6.2	0.560
SLEDAI-2K	10.3 ± 3.7	11.7 ± 4.0	10.2 ± 3.7	**<0.001**
SDI score	0.6 ± 1.1	0.3 ± 0.6	0.6 ± 1.1	**<0.001**
SDI score >0	1146 (35.6%)	44 (22.9%)	1102 (36.4%)	**<0.001**
BILAG renal A	46 (1.4%)	19 (9.9%)	27 (0.9%)	**<0.001**
BILAG renal B	481 (14.9%)	76 (39.6%)	405 (13.4%)	**<0.001**
BILAG renal C	903 (28.0%)	72 (37.5%)	831 (27.4%)	**0.003**
BILAG renal D	331 (10.3%)	14 (7.3%)	317 (10.5%)	0.162
BILAG renal E	1464 (45.4%)	11 (5.7%)	1453 (47.9%)	**<0.001**
BILAG A − D	1761 (54.6%)	181 (94.3%)	1580 (52.1%)	**<0.001**
SLEDAI-2K haematuria	160 (5.0%)	29 (15.1%)	131 (4.2%)	**<0.001**
SLEDAI-2K proteinuria	791 (24.5%)	132 (68.8%)	659 (21.7%)	**<0.001**
SLEDAI-2K pyuria	69 (2.1%)	14 (7.3%)	55 (1.8%)	**<0.001**
SLEDAI-2K urinary casts	16 (0.5%)	3 (1.6%)	13 (0.4%)	0.066
Treatment at baseline				
Glucocorticoid use	2869 (89.0%)	2687 (88.6%)	182 (94.8%)	**0.011**
Prednisone (or equivalent) average dose	12.1 ± 9.4	14.3 ± 11.0	11.9 ± 9.3	**0.003**
Antimalarial agents^b^	2173 (67.4%)	11 (60.4%)	2057 (67.8%)	**0.039**
Immunosuppressants^c^				
AZA	621 (19.3%)	33 (17.2%)	588 (19.4%)	0.512
MTX	366 (11.3%)	16 (8.3%)	350 (11.5%)	0.215
MMFor sodium	509 (15.8%)	58 (30.2%)	451 (14.9%)	**<0.001**
Trial intervention				
Placebo	1077 (33.4%)	83 (43.2%)	994 (32.8%)	**0.003**
Belimumab				
i.v. 1 mg/kg (every fourth week)	559 (17.3%)	14 (7.3%)	545 (18.0%)	**<0.001**
i.v. 10 mg/kg (every fourth week)	1033 (32.0%)	69 (35.9%)	964 (31.8%)	0.232
s.c. 200 mg (weekly)	556 (17.2%)	26 (13.5%)	530 (17.5%)	0.162
**Serological markers at baseline**				
C3; mg/dl	91.8 ± 31.2	73.4 ± 27.1	92.9 ± 31.0	**<0.001**
C4; mg/dl	16.0 ± 9.2	13.3 ± 9	16.1 ± 9.2	**<0.001**
anti-dsDNA; IU/ml	396 ± 1010	518 ± 909	388 ± 1010	0.058
BAFF; µg/l	1.6 ± 1.6	1.6 ± 1.3	1.6 ± 1.6	0.945
Proteinuria; g/24 h	0.6 ± 1	1.6 ± 1.6	0.5 ± 0.9	**<0.001**
eGFR; ml/min	110 ± 35.6	115 ± 42.8	110 ± 35.1	0.106
Creatinine; µmol/l	68 ± 20.5	69.2 ± 34.6	67.9 ± 19.3	0.617
Albumin; g/l	39.7 ± 4.9	35.1 ± 5.4	40 ± 4.8	**<0.001**
Low C3^d^	1621 (50.3%)	141 (73.4%)	1480 (49%)	**<0.001**
Low C4^e^	1377 (42.7%)	91 (47.4%)	1286 (42.4%)	0.200
anti-dsDNA (+)^f^	2336 (72.4%)	162 (84.4%)	2174 (71.7%)	**<0.001**
anti-Sm (+)^g^	776 (30.9%)	43 (47.8%)	733 (30.3%)	**<0.001**
anti-ribosomal P protein (+)^h^	499 (20.2%)	30 (33.7%)	469 (19.7%)	**0.002**
aCL any (+)	637 (20.7%)	32 (19.0%)	605 (20.8%)	0.665
aCL IgA (+)^i^	61 (2.0%)	1 (0.6%)	60 (2.1%)	0.257
aCL IgG (+)^j^	466 (15.1%)	18 (10.7%)	448 (15.3%)	0.129
aCL IgM (+)^k^	254 (8.2%)	27 (10.1%)	237 (8.1%)	0.438

Data are presented as numbers (percentage), mean ± S.D., or median (interquartile range), as appropriate. Statistically significant *P* values are in bold.

a Alaska Native or American Indian from North, South or Central America.

b HCQ, chloroquine, mepacrine, mepacrine hydrochloride or quinine sulphf.

c AZA, CSA, oral CYC, LEF, MTX, mizoribine, MMF, mycophenolate sodium or thalidomide. Cut-off for low complement levels:

d C3 < 90 mg/dl;

e C4 < 16 mg/dl in BLISS-52 and BLISS-76; and <10 mg/dl in BLISS-SC and BLISS-NEA. Cut-off for antibody positivity:

f anti-dsDNA ≥30 IU/ml;

g anti-Sm ≥15 U/ml;

h anti-ribosomal P protein >25 EU/ml;

i IgA aCL IgA ≥15 APL U/ml in BLISS-52 and BLISS-76; and ≥11 APL U/ml in BLISS-SC and BLISS-NEA;

j IgG aCL ≥10 GPL U/ml in BLISS-52 and BLISS-76; and ≥14 GPL U/ml in BLISS-SC and BLISS-NEA;

k IgM aCL ≥10 MPL U/ml in BLISS-52 and BLISS-76; and ≥12 MPL U/ml in BLISS-SC and BLISS-NEA.

SLEDAI-2K: SLEDAI 2000; SDI: SLICC/ACR damage index; C3: complement component 3; C4: complement component 4; BAFF: B cell activating factor belonging to the TNF family; eGFR: estimated glomerular filtration rate; Sm: Smith.

Baseline characteristics of study participants with current or former renal involvement (renal BILAG A–D) and participants with no previous renal involvement (renal BILAG E) are shown in Supplementary Tables S1 and S2, respectively (available at *Rheumatology* online). Baseline characteristics of patients receiving belimumab or placebo including subdivision by current/former renal involvement is presented in Supplementary Table S3 (available at *Rheumatology* online).

### Clinical features in relation to renal flares

Results from Cox regression models are detailed in Supplementary Tables S4–S16 (available at *Rheumatology* online). In univariable Cox regression analysis of the entire study population, patients who developed at least one renal flare had lower extra-renal clinical SLEDAI-2K scores compared with patients who did not develop renal flares (HR: 0.9; 95% CI: 0.8–0.9; *P* < 0.001); this association remained significant after adjustments (Supplementary Table S10, available at *Rheumatology* online). Similar patterns were seen in subgroup analyses, with the exception of belimumab-treated patients in whom the association ceased after adjustments (Supplementary Tables S13 and S14, available at *Rheumatology* online).

Lower proportions among patients who developed renal flares had established organ damage at baseline compared with patients who did not develop renal flares (HR: 0.6; 95% CI: 0.5–0.8; *P* < 0.001); this association remained significant after adjustments (HR: 0.7; 95% CI: 0.6–0.9; *P* = 0.008; Supplementary Table S11, available at *Rheumatology* online). Similar results were documented in subgroup analyses, except for the belimumab-treated subgroup of patients in whom the association ceased after adjustments (Supplementary Tables S13 and S14, available at *Rheumatology* online).

Compared with placebo, use of belimumab in multivariable Cox regression models yielded a 40% reduction in the hazard of renal flare development, both in the pooled study population (HR: 0.6; 95% CI: 0.5–0.8; *P* = 0.002; Supplementary Table S11, available at *Rheumatology* online) and the subgroup with renal BILAG A–D at baseline (HR: 0.6; 95% CI: 0.5–0.9; *P* = 0.003; Supplementary Table S12, available at *Rheumatology* online).

### Clinical chemistry and traditional serological markers

**Figure 1.**
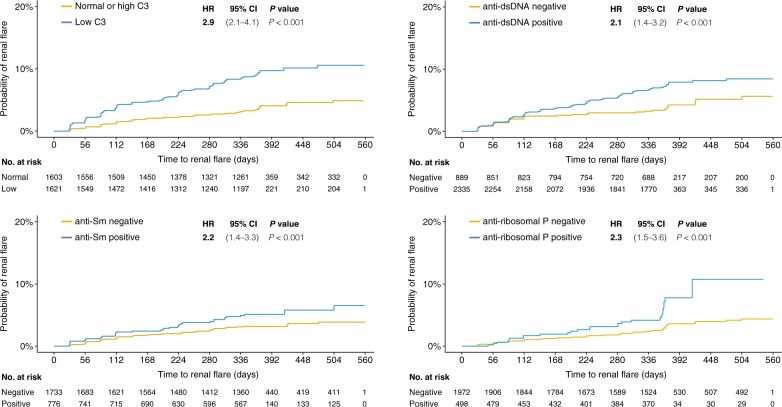
Renal flares over time. The figure depicts the time to renal flare in distinct patient subgroups i.e. patients with low levels of C3 at baseline compared with patients with normal/high C3 levels (A), patients with positive anti-dsDNA antibody levels compared with patients who never tested positive for anti-dsDNA during follow-up (B), patients with positive anti-Sm antibody levels compared with patients who never tested positive for anti-Sm during follow-up (C), and patients with positive anti-ribosomal P protein antibody levels compared with patients who never tested positive for anti-ribosomal P protein autoantibodies during follow-up (**D**). Hazard ratios (HRs) and 95% CIs derive from univariable proportional hazards (Cox) regression analysis. C3: complement component 3; Sm: Smith

**Figure 2.**
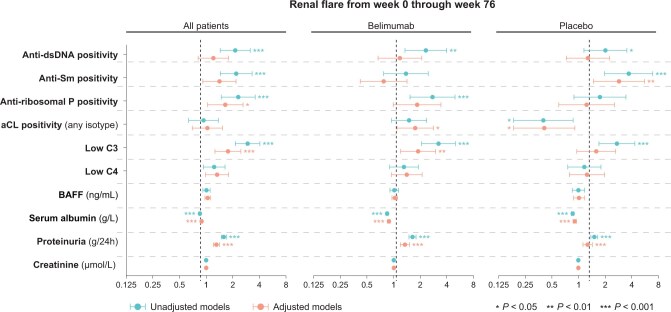
Traditional markers as predictors of renal flares in patients treated with non-biologic standard therapy with or without add-on belimumab. Forest plots illustrating results from univariable and multivariable proportional hazards (Cox) regression analysis. Circles represent hazard ratios (HRs), and whiskers denote the 95% CI. Asterisks denote statistically significant *P* values. BAFF: B cell activating factor belonging to the TNF family; C3: complement component 3; C4: complement component 4; Sm: Smith

In the pooled study population, serum albumin (HR 0.9; 95% CI: 0.8–0.9; *P* < 0.001), proteinuria (HR: 1.6; 95% CI: 1.5–1.7; *P* < 0.001), and low C3 levels (HR: 2.9; 95% CI: 2.1–4.1; *P* < 0.001) were robust determinants of subsequent renal flare occurrence, with the associations remaining significant after adjustments in multivariable models (Supplementary Table S11, available at *Rheumatology* online; Figs 1 and 2). Similar associations were found in subgroup analyses, with the exception of low C3 in the subgroup analysis of patients receiving placebo, where the association attenuated after adjustments (Supplementary Tables S15 and S16, available at *Rheumatology* online). No association was gleaned between low C4 levels and renal flare occurrence, with the exception of an association documented in the subgroup analysis of belimumab-treated patients and only after adjustments (HR: 1.6; 95% CI: 1.0–2.4; *P* = 0.032). Levels of serum creatinine were also found to be predictive of impending renal flares, albeit only in patients with current or former renal involvement at baseline (renal BILAG A–D) and only in the adjusted Cox regression model (HR: 1.0; 95% CI: 1.0–1.0; *P* = 0.020).

We observed an association between anti-dsDNA positivity and renal flare development in the pooled study population (HR: 2.1; 95% CI: 1.4–3.2; *P* < 0.001), which however attenuated in multivariable regression analysis (Fig. 2; Supplementary Table S11, available at *Rheumatology* online). Similar associations between anti-dsDNA positivity and impending renal flare were seen in the subgroup analyses of belimumab-treated patients (Supplementary Table S7, available at *Rheumatology* online) and placebo recipients (Supplementary Table S9, available at *Rheumatology* online), which, again, attenuated after adjustments (Supplementary Tables S13 and S15, available at *Rheumatology* online). No association between anti-dsDNA positivity and renal flares was documented in the subgroup analysis of patients with renal BILAG A–D at baseline (Supplementary Table S5, available at *Rheumatology* online).

Positive levels of anti-Sm antibodies were associated with renal flare occurrence (HR: 2.2; 95% CI: 1.4–3.3; *P* < 0.001); however, the association attenuated after adjustments (Supplementary Table S10, available at *Rheumatology* online). Similar patterns were seen in the subgroup analysis of patients with current or former renal involvement (renal BILAG A–D; Supplementary Table S5, available at *Rheumatology* online) and in patients who received placebo (HR: 3.7; 95% CI: 2.0–6.9; *P* < 0.001; Supplementary Table S9, available at *Rheumatology* online), but not in patients who were treated with belimumab (Supplementary Table S7, available at *Rheumatology* online).

Anti-ribosomal P protein antibody positivity was associated with renal flare development in the pooled study population (HR: 2.3; 95% CI: 1.5–3.6; *P* < 0.001); the association remained significant in the adjusted model (Supplementary Table S11, available at *Rheumatology* online). A similar association was found in patients with renal BILAG A–D at baseline (HR: 1.8; 95% CI: 1.2–2.9; *P* = 0.009; Supplementary Table S5, available at *Rheumatology* online), which remained significant after adjustments (Supplementary Table S12, available at *Rheumatology* online). No association between anti-ribosomal P protein antibody positivity was seen in study participants who received placebo (Supplementary Table S9, available at *Rheumatology* online). Among patients who received belimumab, anti-ribosomal P protein antibody positivity was found to be predictive of impending renal flare (HR: 2.8; 95% CI: 1.5–5.0; *P* = 0.001; Supplementary Table S7, available at *Rheumatology* online); a trend towards an association in the same direction was also seen after adjustments (HR: 1.8; 95% CI: 1.0–3.4; *P* = 0.053; Supplementary Table S13, available at *Rheumatology* online).

Positive levels of aCL antibodies (any isotype) were associated with renal flare development in the belimumab subgroup of patients, yet only after adjustments (HR: 1.8; 95% CI: 1.1–2.8; *P* = 0.020; Supplementary Table S13, available at *Rheumatology* online). A similar association was seen in the subgroup analysis of patients with renal BILAG A–D at baseline (HR: 1.8; 95% CI: 1.1–3.0; *P* = 0.014; Supplementary Table S14, available at *Rheumatology* online). In contrast, aCL antibody positivity yielded a negative association with renal flares in the placebo group (HR: 0.4; 95% CI: 0.2–0.9; *P* = 0.021; Supplementary Table S8, available at *Rheumatology* online), which remained significant after adjustments (HR: 0.4; 95% CI: 0.2–0.9; *P* = 0.028; Supplementary Table S15, available at *Rheumatology* online). Similar patterns were seen in subgroup analyses of patients with renal BILAG A–D at baseline (Supplementary Tables S10 and S16, available at *Rheumatology* online).

No association was documented between baseline serum BAFF levels and impending renal flare in the pooled study population (HR: 1.0; 95% CI: 0.9–1.0; *P* = 0.826; Supplementary Table S4, available at *Rheumatology* online) or any of the subgroup analyses (Supplementary Tabless S5–S16, available at *Rheumatology* online).

The subgroup analysis comprising patients with no prior renal involvement until baseline (renal BILAG E) yielded no significant associations between markers under investigation and impending renal flare (Supplementary Table S6, available at *Rheumatology* online). Due to the limited number of renal flares observed during follow-up in this patient subgroup (*N* = 11), we did not employ multivariable analysis.

Results from variable importance analysis are visualized in Supplementary Figs S1–S3 (available at *Rheumatology* online). In conformity with the results from the Cox regression models, proteinuria, serum albumin, low C3, and anti-ribosomal P antibody positivity were among the top predictors of renal flares in the entire study population and the renal BILAG A–D subgroup. Accuracy in predicting renal flares in the renal BILAG E subgroup was limited.

## Discussion

We herein investigated demographic, clinical and laboratory parameters, and traditional serological markers as potential predictors of renal flares in patients receiving non-biologic ST plus belimumab or placebo for active SLE, yet no current severe renal activity. Previous reports suggest that ST does not prevent renal relapses satisfactorily [23, 24], illustrating the urgent need for identifying patient profiles at risk of developing renal flares despite ongoing immunosuppressive therapy, towards individualization of pharmacotherapy for preserving renal function. We observed that high levels of proteinuria, low serum albumin, and low C3 levels at baseline were robust determinants of renal flares. We also identified factors whose predictive ability differed depending on whether add-on belimumab or placebo was administered. Whereas anti-Sm positivity was associated with renal flares in patients receiving ST alone, anti-ribosomal P antibody positivity was associated with renal flares in the group receiving ST plus belimumab.

Patients who developed at least one renal flare during follow-up were younger compared with patients who did not develop renal flares, which is in line with established knowledge that renal disease preferentially evolves early during disease evolution [2]. Moreover, Asian origin was associated with impending renal flares during follow-up, which is concordant with established knowledge of LN incidence, features, and severity across ethnic groups, with SLE patients of Asian, African, and Hispanic origin being more prone to developing renal disease than Caucasian SLE patients [2, 25]. We documented lower extra-renal disease activity and lower proportions of patients with established organ damage among patients who developed renal flares, the latter partly also reflecting that patients who developed renal flares were younger. Furthermore, and not unexpectedly, patients with current or former renal involvement showed a >9-fold increased hazard for developing a new renal flare in this study.

Moreover, patients on background immunosuppressant therapy with MMF or sodium showed an increased risk for renal flare development, in conformity with a recent report focusing on *de novo* renal flares [26]. The differences between users and non-users of mycophenolic acid observed herein in unadjusted comparisons are likely explained by confounding by indication; MMF (or sodium) is the drug most commonly used in the maintenance phase of LN therapy. This is well-aligned with the finding that BILAG A–D at baseline was a strong predictor of impending renal flare. It must be stressed that the present study was not designed to estimate the effect of background immunosuppressive therapy. As patients enrolled in the BLISS programmes were randomized to receive add-on belimumab or placebo on top of background immunosuppressive therapies that they were on for various amounts of time prior to the study baseline, a specific study design would be required to determine the effect of background medications, accounting for systematic differences between users and non-users.

In the pooled study population, baseline serum albumin, proteinuria, and low C3 levels were robust determinants of subsequent renal flare occurrence; similar associations were found in the belimumab and placebo subgroups as well as in patients with current or previous renal involvement. While proteinuria is used in clinical routine for the surveillance of LN [12], hypoalbuminaemia is not established as an indicator of renal activity in patients with SLE. Hypoalbuminaemia is nevertheless partially a result of proteinuria and has been postulated to be associated with LN relapses [23] and as a marker of SLE activity, particularly renal activity [27]. In the present investigation, we observed a strong inverse association between serum albumin levels and impending renal flare, which remained significant after adjustments. While this was beyond the scope of the present study, investigation of variations over time in biomarker levels as predictors of clinical response and flares as a complement to baseline levels would have merit, as proposed in previous research [28, 29].

While low C3 was herein demonstrated to be a robust determinant of impending renal flares, no clear predictive properties were documented for C4. Similarly, earlier investigations have shown associations between isolated C3 hypocomplemaentemia and ESKD or death [30]. Consistent with our findings, despite divergence in biological phenomena between the target organs and the periphery to a certain extent, C3b- but not C4d-deposition in the kidney has been shown to be associated with progression of renal disease [31, 32]. The C3, C4, and downstream components of the complement cascade are postulated to have central roles in the pathogenic processes in SLE, and their role as biomarkers in LN has been extensively investigated [30, 33, 34]. While C3 and C4 are widely used in SLE and LN surveillance, our study further supports their differential utility as biomarkers of impending renal activity.

We observed an association between anti-dsDNA positivity and renal flare development in univariable models, which however attenuated after adjustments. This association is in line with previous literature; anti-dsDNA antibodies have been postulated to be involved in the pathogenesis of SLE and LN and are widely used in clinical routine as a surveillance tool for disease activity [9, 35]. Furthermore, positive levels of anti-Sm antibodies were associated with renal flare occurrence in patients who received placebo but not in patients who received belimumab. This finding is of interest in light of reports of anti-Sm antibodies being associated with the development of renal flares, and a profound correlation between anti-Sm antibody levels and proteinuria in a previous study [36]. Moreover, a connection between anti-Sm antibodies and LN pathogenesis was postulated in another study [35]. Importantly, the lack of association in the belimumab-treated population can be assumed to be due to a greater benefit conferred from belimumab in anti-Sm–positive individuals, as evidenced in a previous real-world investigation [37].

Anti-ribosomal P protein antibody positivity was associated with renal flare development in belimumab-treated but not in placebo-treated patients. Previous literature has been inconsistent regarding the role of anti-ribosomal P protein antibodies in LN [38, 39]. A previous study provided implications that anti-ribosomal P protein antibodies may be associated with membranous LN, and that simultaneous presence of anti-ribosomal P protein and anti-dsDNA antibodies may signify membranous LN with concomitant proliferative lesions [38]. By contrast, another study suggested that anti-ribosomal P protein antibodies in combination with anti-dsDNA may serve as a marker signifying protection against renal involvement [39]. Even though further investigation is needed regarding the presence of anti-ribosomal P protein antibodies in patients with SLE in relation to kidney involvement, our findings point towards predictive properties for these autoantibodies, which may prove useful in monitoring renal flare development, especially in patients starting belimumab therapy.

Positive baseline levels of aCL antibodies predicted renal flare development in patients treated with belimumab but were protective against renal flares in patients who received placebo. While differential and rather limited aCL data availability across different analyses may partially lie behind this inconsistent result between the two groups of patients, it is worth mentioning that venous thrombosis has previously been linked with reduced belimumab efficacy both in a real-world setting [40] and in clinical trial populations [41]. Moreover, belimumab has been shown to induce reductions of aCL levels only in the presence of antimalarial agents [42]. Besides, aPLs have been associated with renal function diminutions during a renal nephritis flare, but no clear association with long-term renal prognosis has been established [43], suggesting transient reactivity rather than a pathogenic role in renal SLE. Collectively, our observations lend indirect support to the notion that the benefit conferred from belimumab is attenuated in the presence of aPLs or coexisting APS [40, 41], but no firm conclusions can be drawn regarding the value of aPLs in the surveillance of renal activity in SLE.

Serum BAFF levels yielded no association with impending renal flares in the present study. In an earlier work, serum BAFF levels were elevated at the time of active LN as well as after therapy compared with levels in population-based non-SLE controls [44]. Moreover, serum BAFF levels have been reported to be correlated with global SLE disease activity [45–47], as well as renal and CNS activity [48]. The BAFF-var genotype, a genetic variant of the BAFF encoding gene TNF ligand superfamily member 13 b (TNFSF13B), which prevents downregulation of BAFF expression [49], has been associated with the occurrence of LN [50]. Collectively, accumulating evidence suggests that BAFF is a key player in SLE pathogenesis, but while BAFF levels may be indicative of nephritis, thus of diagnostic value, no ability for predicting impending renal flare development could be inferred in the present study.

Additionally, variable importance analysis confirmed the findings from Cox regression analysis, further supporting the hypothesis that proteinuria, low serum albumin, low C3, and the presence of anti-ribosomal P antibodies represent strong predictors of impending renal flare in patients with SLE. The low accuracy seen in the subgroup of patients with no previous renal involvement was likely due to the low number of *de novo* renal flares.

Several limitations must be acknowledged. First, the BLISS trials were not designed to investigate markers of renal flare development. The study participants were selected based on strict inclusion criteria to ascertain homogeneity in the trial populations, which limits the generalizability of the results from the present analyses to the global SLE population. Second, the low number of renal events, particularly in the subgroup of patients with no renal involvement until baseline, likely owing to the stringent definition of renal flare, limited the statistical power in stratified analyses. Nevertheless, distinction between these two patient subgroups was not the main scope of this work, as there were no a priori assumptions of a differential importance of the predictors under investigation in the two subgroups. Moreover, some autoantibodies under investigation were not available in all trials, further decreasing statistical power. Lastly, batch effects across the trials may have introduced bias, which was partially addressed through the use of autoantibody positivity rather than continuous autoantibody levels. Nevertheless, a major strength was the use of data from four large clinical trials, which together constitute one of the largest and most diverse SLE populations studied hitherto for factors associated with renal flare development.

## Concluding remarks

High baseline proteinuria levels, hypoalbuminaemia, and C3 consumption were associated with renal flare development in this clinical trial setting comprising patients with active SLE yet no severe ongoing renal disease. In addition to anti-dsDNA, anti-ribosomal P protein antibody positivity may prove a valuable early signal of impending renal flares in belimumab-treated patients, whereas anti-Sm antibody positivity may predict renal flares in patients treated with non-biologic standard therapy. While low C3 and positive anti-dsDNA levels constitute serological features of known importance in the surveillance of renal SLE, levels of serum albumin, anti-Sm antibodies, and anti-ribosomal P protein antibodies are less established biomarkers in SLE. Further investigation of the predictive properties of these traditional markers as well as combined biomarker panels in SLE populations has merit.

## Supplementary Material

keae023_Supplementary_Data

## Data Availability

The datasets used and analysed during the current study can be made available through the Clinical Study Data Request (CSDR) consortium.
