# Supplementary material for: Predictors of renal flares in systemic lupus erythematosus: a post-hoc analysis of four phase III clinical trials of belimumab
Source: Rheumatology (Oxford). 2024 Jan 12;64(2):623–31. doi: 10.1093/rheumatology/keae023 (PMC11781576; doi:10.1093/rheumatology/keae023)
Supplement: keae023_Supplementary_Data [file keae023_supplementary_data.docx]

# **Predictors of renal flares in systemic lupus erythematosus: a post-hoc analysis of four phase III clinical trials of belimumab**

Sandra Jägerback^1,2^, Alvaro Gomez^1^, Ioannis Parodis^1,3^

^1^Division of Rheumatology, Department of Medicine Solna, Karolinska Institutet and Karolinska University Hospital, Stockholm, Sweden.

^2^Division of Rheumatology, Danderyd University Hospital, Danderyd, Sweden.

^3^Department of Rheumatology, Faculty of Medicine and Health, Örebro University, Örebro, Sweden.

**Table of Contents**

**Supplementary Table S1.** Patient characteristics and comparisons between patients who developed renal flares and patients who did not within patients with current or prior renal involvement (renal BILAG A−D)………………………………………………...………………………………………………...……4

**Supplementary Table S2.** Patient characteristics and comparisons between patients who developed renal flares and patients who did not within patients with no renal involvement until baseline (BILAG E)………………………………………………………………..…………………………………………..6

**Supplementary Table S3.** Patient characteristics stratified by patients receiving belimumab and placebo and by previous renal involvement……………...……………………..........................……………………8

**Supplementary Table S4.** Hazard for renal flare development in the entire study population.....................10

**Supplementary Table S5.** Hazard for renal flare development in patients with current or prior renal involvement (renal BILAG A−D)................................................................................................................11

**Supplementary Table S6.** Hazard for renal flare development in patients with no prior renal involvement (renal BILAG E)...........................................................................................................................................12

**Supplementary Table S7.** Hazard for renal flare development in patients who received belimumab…….13

**Supplementary Table S8.** Hazard for renal flare development in patients with current or prior renal involvement (renal BILAG A−D) who received belimumab........................................................................14

**Supplementary Table S9.** Hazard for renal flare development in the placebo group………………….….15

**Supplementary Table S10.** Hazard for renal flare development in patients with current or prior renal involvement (renal BILAG A−D) who received placebo.............................................................................16

**Supplemental Table S11.** Adjusted hazard for renal flare development in the entire study.............................................................................................................................................................17

**Supplementary Table S12.** Adjusted hazard for renal flare development in patients with current or prior renal involvement (renal BILAG A−D)........................................................................................................18

**Supplementary Table S13.** Adjusted hazard for renal flare development in the belimumab group……....19

**Supplementary Table S14.** Adjusted hazard for renal flare development in patients with current or prior renal involvement (renal BILAG A−D) who received belimumab...............................................................20

**Supplementary Table S15.** Adjusted hazard for renal flare development in patients who received placebo.........................................................................................................................................................21

**Supplementary Table S16.** Adjusted hazard for renal flare in patients with current or prior renal involvement (renal BILAG A−D) who received placebo.............................................................................22

**Supplementary Figure S1.** Relative importance of contributing predictors in the entire study population…………………………………………………………………………………………………23

**Supplementary Figure S2.** Relative importance of contributing predictors in patients with current or prior renal involvement (renal BILAG A−D)……………………………………………………………………24

**Supplementary Figure S3.** Relative importance of contributing predictors in patients with no prior renal involvement (renal BILAG E)…………………………………….………………………………25

**Supplementary Table S1.** Patient characteristics and comparisons between patients who developed renal flares and patients who did not within patients with current or prior renal involvement (renal BILAG A−D).

|  | All patients | Renal flare | No renal flare | P value |
| --- | --- | --- | --- | --- |
|  | **N=1761** | **N=181** | **N=1580** |  |
| Patient characteristics | | | | |
| Age at baseline (years) | 34.8 ± 10.9 | 31.8 ±10.4 | 35.1 ± 10.9 | **<0.001** |
| BMI; kg/m² | 24.1 ± 5.3 | 23.4 ± 4.7 | 24.2 ± 5.4 | **0.032** |
| Female sex | 1630 (92.6%) | 164 (90.6%) | 1466 (92.8%) | 0.364 |
| Ancestry |  |  |  |  |
| Asian | 950 (53.9%) | 135 (74.6%) | 815 (51.6%) | **<0.001** |
| Black/African American | 112 (6.4%) | 9 (5.0%) | 103 (6.5%) | 0.419 |
| Indigenous American* | 213 (12.1%) | 14 (7.7%) | 199 (12.6%) | 0.058 |
| White/Caucasian | 486 (27.6%) | 23 (12.7%) | 463 (29.3%) | **<0.001** |
| Clinical data | | | | |
| SLE duration at baseline (years) | 6.5 ± 6.1 | 6.1 ± 5.5 | 6.5 ± 6.1 | 0.355 |
| SLEDAI-2K | 11.0 ± 4.0 | 11.8 ±4.1 | 10.9 ± 4.0 | **0.004** |
| SDI score | 0.6 ± 1.0 | 0.3 ± 0.6 | 0.6 ± 1.1 | **<0.001** |
| SDI score >0 | 581 (33.0%) | 41 (22.7%) | 540 (34.2%) | **0.002** |
| BILAG renal A | 46 (2.6%) | 19 (10.5%) | 27 (1.7%) | **<0.001** |
| BILAG renal B | 481 (27.3%) | 76 (42.0%) | 405 (25.6%) | **<0.001** |
| BILAG renal C | 903 (51.3%) | 72 (39.8%) | 831 (52.6%) | **0.001** |
| BILAG renal D | 331 (18.8%) | 14 (7.7%) | 317 (20.1%) | **<0.001** |
| BILAG renal E | NA | NA | NA | NA |
| BILAG renal A−D | NA | NA | NA | NA |
| SLEDAI-2K haematuria | 152 (8.6%) | 29 (16.0%) | 123 (7.8%) | **<0.001** |
| SLEDAI-2K proteinuria | 768 (43.6%) | 132 (72.9%) | 636 (40.3%) | **<0.001** |
| SLEDAI-2K pyuria | 66 (3.8%) | 14 (7.7%) | 52 (3.3%) | **0.006** |
| SLEDAI-2K urinary casts | 16 (0.9%) | 3 (1.7%) | 13 (0.8%) | 0.222 |
| Treatment at baseline |  |  |  |  |
| Glucocorticoids use | 1646 (93.5%) | 174(96.1%) | 1472 (93.2%) | 0.170 |
| Prednisone (or equivalent) average dose | 13.7 ± 9.8 | 14.6 ± 11.1 | 13.6 ± 9.7 | 0.218 |
| AMA^†^ | 1209 (68.7%) | 111 (61.3%) | 1198 (69.5%) | **0.027** |
| Immunosuppressants^‡^ |  |  |  |  |
| Azathioprine | 334 (19.0%) | 31 (17.1%) | 303 (19.2%) | 0.571 |
| Methotrexate | 142 (8.1%) | 15 (8.3%) | 127 (8.0%) | 1.000 |
| Mycophenolate mofetil or sodium | 403 (22.9%) | 57 (31.5%) | 346 (21.9%) | **0.005** |
| Trial intervention |  |  |  |  |
| Placebo | 604 (34.3%) | 78 (43.1%) | 526 (33.3%) | **0.008** |
| Belimumab |  |  |  |  |
| i.v. 1 mg/kg (every 4^th^ week) | 238 (13.5%) | 13 (7.2%) | 225 (14.2%) | **0.009** |
| i.v. 10 mg/kg (every 4^th^ week) | 630 (35.8%) | 67 (37.0%) | 563 (35.6%) | 0.713 |
| s.c. 200 mg (weekly) | 289 (16.4%) | 23 (12.7%) | 266 (16.8%) | 0.155 |
| Serological markers at baseline | | | | |
| C3; mg/dL | 84.7 ± 29.6 | 72.4 ± 26.7 | 86.1 ± 29.6 | **<0.001** |
| C4; mg/dL | 15.2 ± 9.0 | 13.1 ± 9.1 | 15.4 ± 9.0 | **0.002** |
| anti-dsDNA; IU/mL | 517 ± 1210 | 537 ± 932 | 514 ± 1240 | 0.770 |
| BAFF; µg/L | 1.6 ± 1.9 | 1.6 ± 1.3 | 1.6 ± 1.9 | 0.640 |
| Proteinuria; g/24 h | 0.9 ± 1.3 | 1.7 ± 1.6 | 0.8 ± 1.2 | **<0.001** |
| eGFR; mL/min | 110 ± 37.0 | 115 ± 43.6 | 109 ± 36.1 | 0.055 |
| Creatinine; µmol/L | 68.6 ± 24.7 | 69.2 ± 34.9 | 68.5 ± 23.3 | 0.783 |
| Albumin; g/L | 38.2 ± 5.3 | 34.7 ± 5.3 | 38.6 ± 5.2 | **<0.001** |
| Low C3^G^ | 1046 (59.4%) | 135 (34.6%) | 911 (57.7%) | **<0.001** |
| Low C4^H^ | 771 (43.8%) | 88 (48.6%) | 683 (43.2%) | 0.192 |
| anti-dsDNA (+)^A^ | 1420 (80.6%) | 154 (85.1.6%) | 1266 (80.1%) | 0.134 |
| anti-Sm (+)^B^ | 455 (39.4%) | 41 (50.0%) | 414 (38.5%) | 0.054 |
| anti-ribosomal P protein (+)^C^ | 291 (25.4%) | 29 (35.8%) | 262 (24.6%) | **0.037** |
| aCL any (+) | 328 (19.7%) | 30 (19.0%) | 298 (19.8%) | 0.892 |
| aCL IgA (+)^D^ | 36 (2.2%) | 1 (0.6%) | 35 (2.3%) | 0.248 |
| aCL IgM (+)^E^ | 126 (7.6%) | 16 (10.1%) | 110 (7.3%) | 0.260 |
| aCL IgG (+)^F^ | 239 (14.3%) | 17 (10.8%) | 222 (14.7%) | 0.219 |

Data are presented as numbers (percentage), mean ± standard deviation, or median (interquartile range), as appropriate. Statistically significant P values are in bold. BMI: body mass index; SLE: systemic lupus erythematosus; SLEDAI-2K: Systemic Lupus Erythematous Disease Activity Index 2000; SDI: SLICC/ACR damage index; BILAG: British Isles Lupus Assessment Group; AMA: antimalarial agents; i.v.: intravenous; s.c.: subcutaneous; C3: complement component 3; C4: complement component 4; BAFF: B cell activating factor belonging to the TNF family; eGRF: estimated glomerular filtration rate; Sm: Smith; aCL: anticardiolipin antibody; Ig immunoglobulin.

*Alaska Native or American Indian from North, South or Central America.

^†^Hydroxychloroquine, chloroquine, mepacrine, mepacrine hydrochloride or quinine sulfate.

^‡^Azathioprine, cyclosporine, oral cyclophosphamide, leflunomide, methotrexate, mizoribine, mycophenolate mofetil, mycophenolate sodium or thalidomide.

**Cut-off for low complement levels:**

^A^C3 <90 mg/dL

^B^C4 <16 mg/dL in BLISS-52 and BLISS-76 and <10 mg/dL in BLISS-SC and BLISS-NEA

**Cut-off for antibody positivity:**

^C^anti-dsDNA ≥30 IU/mL

^D^anti-Sm ≥15 U/mL

^E^anti-ribosomal P protein >25 EU/mL

^F^IgA aCL IgA ≥15 APL U/mL in BLISS-52 and BLISS-76 and ≥11 APL U/mL in BLISS-SC and BLISS-NEA

^G^IgG aCL ≥10 GPL U/mL in BLISS-52 and BLISS-76 and ≥14 GPL U/mL in BLISS-SC and BLISS-NEA

^H^IgM aCL ≥10 MPL U/mL in BLISS-52 and BLISS-76 and ≥12 MPL U/mL in BLISS-SC and BLISS-NEA

**Supplementary Table S2.** Patient characteristics and comparisons between patients who developed renal flares and patients who did not within patients with no renal involvement until baseline (BILAG E).

|  | All patients | Renal flare | No renal flare | P value |
| --- | --- | --- | --- | --- |
|  | **N=1464** | **N=11** | **N=1453** |  |
| Patient characteristics | | | | |
| Age at baseline (years) | 39.1 ± 11.9 | 37.9 ± 13.1 | 39.1 ± 11.9 | 0.771 |
| BMI; kg/m² | 26.0 ± 6.3 | 24.5 ± 5.3 | 26.0 ± 6.3 | 0.366 |
| Female sex | 1400 (95.6%) | 11 (100%) | 1389 (95.6%) | 1.000 |
| Ancestry |  |  |  |  |
| Asian | 292 (19.9%) | 3 (27.3%) | 289 (19.9%) | 0.467 |
| Black/African American | 122 (8.3%) | 0 (0%) | 122 (8.4%) | 0.614 |
| Indigenous American* | 236 (16.1%) | 2 (18.2%) | 234 (16.1%) | 0.694 |
| White/Caucasian | 814 (55.6%) | 6 (54.5%) | 808 (55.6%) | 1.000 |
| Clinical data | | | | |
| SLE duration at baseline (years) | 6.3 ± 6.4 | 6.9 ± 5.4 | 6.3 ± 6.4 | 0.697 |
| SLEDAI-2K | 9.5 ± 3.1 | 9.8 ± 2.9 | 9.5 ± 3.1 | 0.737 |
| SDI score | 0.7 ± 1.2 | 0.4 ± 0.7 | 0.7 ± 1.2 | 0.131 |
| SDI score >0 | 565 (38.6) | 3 (27.3) | 562 (38.7) | 0.545 |
| SLEDAI-2K haematuria | 8 (0.5%) | 0 (0.0%) | 8 (0.6%) | 1.000 |
| SLEDAI-2K proteinuria | 23 (1.6%) | 0 (0.0%) | 23 (1.6%) | 1.000 |
| SLEDAI-2K pyuria | 3 (0.2%) | 0 (0.0%) | 3 (0.2%) | 1.000 |
| SLEDAI-2K urinary casts | 0 (0.0%) | 0 (0.0%) | 0 (0.0%) | NA |
| Treatment at baseline |  |  |  |  |
| Glucocorticoid use | 1223 (83.5%) | 8 (72.7%) | 1215 (83.6%) | 0.404 |
| Prednisone (or equivalent) average dose | 10.1 ± 8.4 | 9.8 ± 8.8 | 10.1 ± 8.4 | 0.891 |
| AMA^†^ | 96 (65.8%) | 5 (45.4%) | 959 (66.0%) | 0.152 |
| Immunosuppressants^‡^ |  |  |  |  |
| Azathioprine | 287 (19.6%) | 2 (18.2%) | 285 (19.6%) | 1.000 |
| Methotrexate | 224 (15.3%) | 1 (9.1%) | 223 (15.3%) | 1.000 |
| Mycophenolate mofetil or sodium | 106 (7.2%) | 1 (9.1%) | 105 (7.2%) | 0.564 |
| Trial intervention |  |  |  |  |
| Placebo | 473 (32.3%) | 5 (45.5%) | 468 (32.2%) | 0.349 |
| Belimumab |  |  |  |  |
| i.v. 1 mg/kg (every 4^th^ week) | 321 (21.9%) | 1 (9.1%) | 320 (22.0%) | 0.473 |
| i.v. 10 mg/kg (every 4^th^ week) | 403 (27.5%) | 2 (18.2%) | 401 (27.6%) | 0.737 |
| s.c. 200 mg (weekly) | 267 (18.2%) | 3 (27.3%) | 264 (18.2%) | 0.432 |
| Serological markers at baseline | | | | |
| C3; mg/dL | 100 ± 30.9 | 89.8 ± 29.8 | 100 ± 30.9 | 0.268 |
| C4; mg/dL | 17 ± 9.4 | 17 ± 7.0 | 17 ± 9.4 | 0.986 |
| anti-dsDNA; IU/mL | 250 ± 664 | 212 ± 234 | 251 ± 666 | 0.604 |
| BAFF; µg/L | 1.5 ± 1.1 | 1.5 ± 1.3 | 1.5 ± 1.1 | 0.877 |
| Proteinuria; g/24 h | 0.1 ± 0.2 | 0.2 ± 0.1 | 0.1 ± 0.2 | 0.524 |
| eGFR; mL/min | 111 ± 33.9 | 106 ± 27.2 | 111 ± 33.9 | 0.615 |
| Creatinine; µmol/L | 67.3 ± 13.8 | 68.3 ± 28.7 | 67.3 ± 13.7 | 0.906 |
| Albumin; g/L | 41.6 ± 3.7 | 40.2 ± 3.1 | 41.6 ± 3.7 | 0.161 |
| Low C3^G^ | 575 (39.3%) | 6(54.5%) | 569 (39.2%) | 0.298 |
| Low C4^H^ | 604 (41.4%) | 3 (27.3%) | 603 (41.5%) | 0.541 |
| anti-dsDNA(+)^A^ | 916 (62.6%) | 8 (72.7%) | 908 (62.5%) | 0.552 |
| anti-Sm(+)^B^ | 321 (23.7%) | 2 (25%) | 319 (23.7%) | 1.000 |
| anti-Ribosomal P protein (+)^C^ | 208 (15.7%) | 1 (12.5%) | 207 (15.7%) | 1.000 |
| aCL any (+) | 309 (21.8%) | 2 (20%) | 307 (21.8%) | 1.000 |
| aCL IgA (+)^D^ | 25 (1.8%) | 0 (0%) | 25 (1.8%) | 1.000 |
| aCL IgM(+)^E^ | 128 (9.2%) | 1 (10%) | 127 (9%) | 0.612 |
| aCL IgG (+)^F^ | 227 (16.0%) | 1 (10%) | 226 (16%) | 1.000 |

Data are presented as numbers (percentage), mean ± standard deviation, or median (interquartile range), as appropriate. Statistically significant P values are in bold. BMI: body mass index; SLE: systemic lupus erythematosus; SLEDAI-2K: Systemic Lupus Erythematous Disease Activity Index 2000; SDI: SLICC/ACR damage index; BILAG: British Isles Lupus Assessment Group; AMA: antimalarial agents; i.v.: intravenous; s.c.: subcutaneous; C3: complement component 3; C4: complement component 4; BAFF: B cell activating factor belonging to the TNF family; eGRF: estimated glomerular filtration rate; Sm: Smith; aCL: anticardiolipin antibody; Ig immunoglobulin.

*Alaska Native or American Indian from North, South or Central America.

^†^Hydroxychloroquine, chloroquine, mepacrine, mepacrine hydrochloride or quinine sulfate.

^‡^Azathioprine, cyclosporine, oral cyclophosphamide, leflunomide, methotrexate, mizoribine, mycophenolate mofetil, mycophenolate sodium or thalidomide.

**Cut-off for low complement levels:**

^A^C3 <90 mg/dL

^B^C4 <16 mg/dL in BLISS-52 and BLISS-76 and <10 mg/dL in BLISS-SC and BLISS-NEA

**Cut-off for antibody positivity:**

^C^anti-dsDNA ≥30 IU/mL

^D^anti-Sm ≥15 U/mL

^E^anti-ribosomal P protein >25 EU/mL

^F^IgA aCL IgA ≥15 APL U/mL in BLISS-52 and BLISS-76 and ≥11 APL U/mL in BLISS-SC and BLISS-NEA

^G^IgG aCL ≥10 GPL U/mL in BLISS-52 and BLISS-76 and ≥14 GPL U/mL in BLISS-SC and BLISS-NEA

^H^IgM aCL ≥10 MPL U/mL in BLISS-52 and BLISS-76 and ≥12 MPL U/mL in BLISS-SC and BLISS-NEA

|  | All patients | | Renal BILAG A−D | | Renal BILAG E | |
| --- | --- | --- | --- | --- | --- | --- |
|  | **Belimumab** | **Placebo** | **Belimumab** | **Placebo** | **Belimumab** | **Placebo** |
|  | **N=2148** | **N=1077** | **N=1157** | **N=604** | **N=991** | **N=473** |
| Patient characteristics | | | | | |  |
| Age at baseline (years) | 36.6 ± 11.4 | 37.1 ± 11.9 | 34.4 ± 10.7 | 35.5 ± 11.3 | 39.1 ± 11.6 | 39.0 ± 12.5 |
| BMI; kg/m² | 25.0 ± 5.9 | 25.0 ± 5.9 | 24.1 ± 5.3 | 24.2 ± 5.4 | 26.0 ± 6.4 | 25.9 ± 6.26 |
| Female sex | 2022 (94.1%) | 1008 (93.6%) | 1073 (92.7%) | 557 (92.2%) | 949 (95.8%) | 451 (95.3%) |
| Ancestry |  |  |  |  |  |  |
| Asian | 828 (38.5%) | 414 (38.4%) | 629 (54.4%) | 321 (53.1%) | 199 (20.1%) | 93 (19.7%) |
| Black/African American | 154 (7.2%) | 80 (7.4%) | 72 (6.2%) | 40 (6.6%) | 82 (8.3%) | 40 (8.5%) |
| Indigenous American* | 449 (13.9%) | 147 (13.6%) | 145 (12.5%) | 68 (11.3%) | 157 (15.8%) | 79 (16.7%) |
| White/Caucasian | 864 (40.2%) | 436 (40.5%) | 311 (26.9%) | 175 (29.0%) | 553 (58.8%) | 261 (55.2%) |
| Clinical data | | | | | | |
| SLE duration at baseline (years) | 6.3 ± 6.1 | 6.6 ± 6.3 | 6.5 ± 6.0 | 6.4 ± 6.1 | 6.0 ± 6.3 | 6.7 ± 6.5 |
| SLEDAI-2K | 10.3 ± 3.7 | 10.3 ± 3.7 | 10.9 ± 4.0 | 11 ± 4.1 | 9.6 ± 3.2 | 9.4 ± 2.9 |
| SDI score | 0.6 ± 1.1 | 0.6 ± 1.1 | 0.5 ± 1.0 | 0.6 ± 1.1 | 0.7 ± 1.2 | 0.7 ± 1.2 |
| SDI score >0 | 754 (35.1%) | 392 (36.4%) | 379 (32.8%) | 202 (33.4%) | 375 (37.9%) | 190 (40.2%) |
| BILAG renal A | 32 (1.5%) | 14 (1.3%) | 32 (2.8%) | 14 (2.3%) | 0% | 0% |
| BILAG renal B | 322 (15.0%) | 159 (14.8%) | 322 (27.8%) | 159 (26.3%) | 0% | 0% |
| BILAG renal C | 582 (27.1%) | 321 (29.8%) | 582 (50.3%) | 321 (53.1%) | 0% | 0% |
| BILAG renal D | 221 (10.3%) | 110 (10.2%) | 221 (19.1%) | 110 (18.2%) | 0% | 0% |
| BILAG renal E | 991 (46.1%) | 473 (43.9%) | 0% | 0% | 100% | 100% |
| BILAG renal A−D | 1157 (53,9%) | 604 (56.1%) | 100% | 100% | 0% | 0% |
| SLEDAI-2K haematuria | 107 (5.0%) | 53 (4.9%) | 102 (8.8%) | 50 (8.3%) | 5 (0.5%) | 3 (0.3%) |
| SLEDAI-2K proteinuria | 527 (24.5%) | 264 (24.5%) | 511 (44.2%) | 257 (42.5%) | 16 (1.6%) | 7 (1.5%) |
| SLEDAI-2K pyuria | 50 (2.3%) | 19 (1.8) | 48 (4.15%) | 18 (3.0%) | 2 (0.2%) | 1 (0.2%) |
| SLEDAI-2K urinary casts | 10 (0.5%) | 6 (0.6%) | 10 (0.9%) | 6 (1.0%) | 0 (0.0%) | 0 (0.0%) |
| Treatment at baseline |  |  |  |  |  |  |
| Glucocorticoids use | 1908 (88.8%) | 961 (89.2%) | 1080 (93.3%) | 566 (93.7%) | 828 (83.6%) | 395 (83.5%) |
| Prednisone (or equivalent)  average dose | 12.0 ± 9.3 | 12.2 ± 9.5 | 13.7 ± 9.9 | 13.6 ± 9.7 | 10.0 ± 8.14 | 10.4 ± 9.1 |
| AMA^†^ | 1440 (67.0%) | 733 (68.1%) | 799 (69.1%) | 410 (67.9%) | 641 (64.7%) | 323 (68.3%) |
| Immunosuppressants^‡^ |  |  |  |  |  |  |
| Azathioprine | 424 (19.7%) | 197 (18.30%) | 227 (19.6%) | 107 (17.7%) | 197 (19.9%) | 90 (19.0%) |
| Methotrexate | 217 (10.1%) | 149 (13.8%) | 81 (7.0%) | 61 (10.1%) | 136 (13.7%) | 88 (18.6%) |
| Mycophenolate mofetil  or sodium | 334 (15.5%) | 157 (16.2%) | 259 (22.4%) | 144 (23.8%) | 75 (7.6%) | 31 (6.6%) |
| Trial intervention |  |  |  |  |  |  |
| Placebo | 0% | 1077 (100%) | 0% | 604 (100%) | 0% | 473 (100%) |
| Belimumab |  |  |  |  |  |  |
| i.v. 1 mg/kg (every 4^th^ week) | 559 (26.0%) | 0% | 238 (20.6%) | 0% | 321 (32.4%) | 0% |
| i.v. 10 mg/kg (every 4^th^ week) | 1033 (48.1%) | 0% | 630 (54.5%) | 0% | 403 (40.7%) | 0% |
| s.c. 200 mg (weekly) | 556 (25.9%) | 0% | 289 (25.0%) | 0% | 267 (26.9%) | 0% |
| Serological markers at baseline | | | | | | |
| C3; mg/dL | 91.5 ± 31.1 | 92.2 ± 31.3 | 84.7 ± 29.7 | 84.5 ± 29.4 | 99.4 ± 30.8 | 100.2 ± 31.0 |
| C4; mg/dL | 15.9 ± 9.2 | 16.0 ± 9.3 | 15.1 ± 8.7 | 15.2 ± 10.0 | 16.9 ± 9.6 | 17.1 ± 8.8 |
| anti-dsDNA; IU/mL | 416 ± 1120 | 356 ± 751 | 557 ± 1350 | 441 ± 878 | 251 ± 720 | 248 ± 529 |
| BAFF; µg/L | 1.6 ± 1.7 | 1.6 ± 1.4 | 1.6 ± 2.0 | 1.7 ± 1.6 | 1.6 ± 1.2 | 1.5 ± 0.9 |
| Proteinuria; g/24 h | 0.6 ± 1.0 | 0.6 ± 1.1 | 0.9 ± 1.2 | 1.0 ± 1.3 | 0.1 ± 0.1 | 0.1 ± 0.2 |
| eGFR; mL/min | 110 ± 35.7 | 110 ± 35.4 | 110 ± 37.0 | 109 ± 37.0 | 111 ± 34.2 | 111 ± 33.2 |
| Creatinine; µmol/L | 68 ± 20.7 | 68 ± 20.1 | 68.6 ± 25.2 | 68.6 ± 23.8 | 67.4 ± 13.8 | 67.2 ± 14.0 |
| Albumin; g/L | 39.7 ± 5.0 | 39.7 ± 4.8 | 38.1 ± 5.4 | 38.3 ± 5.1 | 41.6 ± 3.7 | 41.5 ± 3.7 |
| Low C3^A^ | 1099 (51.2%) | 522 (48.5%) | 703 (60.8%) | 343 (56.8%) | 396 (40.0%) | 179 (37.8% |
| Low C4^B^ | 926 (43.1%) | 451 (41.9%) | 503 (43.5%) | 268 (44.4%) | 423 (42.7%) | 183 (38.7%) |
| anti-dsDNA (+)^C^ | 1578 (73.5%) | 758 (70.4%) | 954 (82.5%) | 466 (77.2%) | 624 (63.0%) | 292 (61.7%) |
| anti-Sm (+)^D^ | 521 (31.2%) | 255 (30.4%) | 306 (40.2%) | 149 (37.7%) | 215 (23.7%) | 106 (23.8%) |
| anti-ribosomal P protein (+)^E^ | 319 (19.4%) | 180 (21.8%) | 189 (25.1%) | 102 (26.2%) | 130 (14.6%) | 78 (17.9%) |
| aCL any (+) | 435 (21.2%) | 202 (19.6%) | 222 (20.3%) | 106 (18.7%) | 213 (22.3%) | 96 (20.7%) |
| aCL IgA (+)^F^ | 37 (1.8%) | 24 (2.3%) | 22 (2.0%) | 14 (2.5%) | 15 (1.6%) | 10 (2.2%) |
| aCL IgG (+)^G^ | 312 (15.2%) | 154 (14.9%) | 162 (14.8%) | 77 (13.5%) | 150 (15.7%) | 77 (16.6%) |
| aCL IgM (+)^H^ | 186 (9.1%) | 68 (6.6%) | 89 (8.1%) | 37 (6.5%) | 97 (10.1%) | 31 (6.7%) |

**Supplementary Table S3.** Patient characteristics stratified by patients receiving belimumab and placebo and by previous renal involvement.

Data are presented as numbers (percentage), mean ± standard deviation, or median (interquartile range), as appropriate. BMI: body mass index; SLE: systemic lupus erythematosus; SLEDAI-2K: Systemic Lupus Erythematous Disease Activity Index 2000; SDI: SLICC/ACR damage index; BILAG: British Isles Lupus Assessment Group; AMA: antimalarial agents; i.v.: intravenous; s.c.: subcutaneous; C3: complement component 3; C4: complement component 4; BAFF: B cell activating factor belonging to the TNF family; eGRF: estimated glomerular filtration rate; Sm: Smith; aCL: anticardiolipin antibody; Ig immunoglobulin.

*Alaska Native or American Indian from North, South or Central America.

^†^Hydroxychloroquine, chloroquine, mepacrine, mepacrine hydrochloride or quinine sulfate.

^‡^Azathioprine, cyclosporine, oral cyclophosphamide, leflunomide, methotrexate, mizoribine, mycophenolate mofetil, mycophenolate sodium or thalidomide.

**Cut-off for low complement levels:**

^A^C3 <90 mg/dL

^B^C4 <16 mg/dL in BLISS-52 and BLISS-76 and <10 mg/dL in BLISS-SC and BLISS-NEA

**Cut-off for antibody positivity:**

^C^anti-dsDNA ≥30 IU/Ml

^D^anti-Sm ≥15 U/mL

^E^anti-ribosomal P protein >25 EU/mL

^F^IgA aCL IgA ≥15 APL U/mL in BLISS-52 and BLISS-76 and ≥11 APL U/mL in BLISS-SC and BLISS-NEA

^G^IgG aCL ≥10 GPL U/mL in BLISS-52 and BLISS-76 and ≥14 GPL U/mL in BLISS-SC and BLISS-NEA

^H^IgM aCL ≥10 MPL U/mL in BLISS-52 and BLISS-76 and ≥12 MPL U/mL in BLISS-SC and BLISS-NEA

**Supplementary Table S4.** Hazard for renal flare development in the entire study population.

|  | Coefficient | HR | 95% CI | P value |
| --- | --- | --- | --- | --- |
| Patient characteristics | | | | |
| Age (years) | 0.0 | 1.0 | 0.9−1.0 | **<0.001** |
| Male sex | 0.4 | 1.5 | 0.9−2.5 | 0.107 |
| Ethnicity (ref.: White/Caucasian) |  |  |  |  |
| African American | 0.6 | 1.9 | 0.9−4.0 | 0.096 |
| Asian | 1.8 | 6.1 | 4.0−9.2 | **<0.001** |
| Indigenous American* | 0.6 | 1.8 | 1.0−3.3 | 0.063 |
| BMI (kg/m²) | -0.1 | 0.9 | 0.9−1.0 | **<0.001** |
| SLE duration | 0.0 | 1.0 | 1.0−1.0 | 0.535 |
| Extra-renal cSLEDAI-2K^‡^ | -0.2 | 0.9 | 0.8−0.9 | **<0.001** |
| SDI score | -0.5 | 0.6 | 0.5−0.8 | **<0.001** |
| Renal BILAG A−D | 2.7 | 15.4 | 8.3−28.2 | **<0.001** |
| anti-dsDNA (+)^A^ | 0.8 | 2.1 | 1.4−3.2 | **<0.001** |
| anti-Sm (+)^B^ | 0.8 | 2.2 | 1.4−3.3 | **<0.001** |
| anti-Ribosomal P protein (+)^C^ | 0.8 | 2.3 | 1.5−3.6 | **<0.001** |
| Any aCL(+) | -0.1 | 0.9 | 0.6−1.4 | 0.738 |
| Low C3^D^ | 1.1 | 2.9 | 2.1−4.1 | **<0.001** |
| Low C4 ^E^ | 0.2 | 1.2 | 0.9−1.6 | 0.147 |
| BAFF | 0.0 | 1.0 | 0.9−1.0 | 0.826 |
| Albumin (g/L) | -0.2 | 0.9 | 0.8−0.9 | **<0.001** |
| Proteinuria (g/24 h) | 0.5 | 1.6 | 1.5−1.7 | **<0.001** |
| Creatinine (µmol/L) | 0.0 | 1.0 | 0.1−1.0 | 0.602 |
| Treatment at baseline |  |  |  |  |
| Prednisone (or equivalent) average dose | 0.0 | 1.0 | 1.0−1.0 | **<0.001** |
| Glucocorticoid use | 0.9 | 2.4 | 1.3−4.6 | **0.006** |
| AMA† | -0.3 | 0.7 | 0.5−1.0 | **0.026** |
| Azathioprine | -0.1 | 0.9 | 0.6−1.3 | 0.524 |
| Mycofenolate mofetil or sodium | 0.9 | 2.4 | 1.7−3.2 | **<0.001** |
| Metothrexate | -0.4 | 0.7 | 0.4−1.1 | 0.148 |
| Trial intervention |  |  |  |  |
| Belimumab |  |  |  |  |
| i.v. 1 mg/kg (every 4^th^ week) | -1.2 | 0.3 | 0.2−0.5 | **<0.001** |
| i.v. 10 mg/kg (every 4^th^ week) | -0.2 | 0.8 | 0.6−1.1 | 0.258 |
| s.c. 200 mg (weekly) | -0.5 | 0.6 | 0.4−1.0 | **0.028** |

Results from univariable proportional hazards (Cox) regression analysis, with data from the pooled BLISS study population. Proteinuria was estimated by 24-hour urine protein excretion, or the urine protein to creatinine ratio (UPCR). Data are presented as the coefficients, hazard ratios (HR), 95% confidence intervals (CI), and P values. Statistically significant P values are in bold. BMI: body mass index; SLE: systemic lupus erythematosus; cSLEDAI-2K: clinical Systemic Lupus Erythematous Disease Activity Index 2000; SDI: SLICC/ACR damage index; BILAG: British Isles Lupus Assessment Group; Sm: Smith; aCL: anticardiolipin antibody; C3: complement component 3; BAFF: B cell activating factor belonging to the TNF family; AMA: antimalarial agents; i.v.: intravenous; s.c.: subcutaneous.

*Alaska Native or American Indian from North, South or Central America.

† Hydroxychloroquine, chloroquine, mepacrine, mepacrine hydrochloride or quinine sulfate.

‡SLEDAI-2K after suppression of serological (anti-dsDNA, C3 and C4) and renal descriptors (proteinuria, haematuria, pyuria and urinary casts).

**Cut-off for antibody positivity:**

^A^anti-dsDNA ≥30 IU/mL

^B^anti-Sm ≥15 U/mL

^C^anti-ribosomal P protein >25 EU/mL

**Cut-off for low complement levels:**

^D^C3 <90 mg/dL

^E^C4 <16 mg/dL in BLISS-52 and BLISS-76 and <10 mg/dL in BLISS-SC and BLISS-NEA

**Supplementary Table S5.** Hazard for renal flare development in patients with current or prior renal involvement (renal BILAG A−D).

|  | Coefficient | HR | 95% CI | P value |
| --- | --- | --- | --- | --- |
| Patient characteristics | | | | |
| Age (years) | 0.0 | 1.0 | 1.0−1.0 | **<0.001** |
| Male sex | 0.3 | 1.3 | 0.8−2.1 | 0.302 |
| Ethnicity (ref.: White/Caucasian) |  |  |  |  |
| African American | 0.6 | 1.8 | 0.8−3.9 | 0.132 |
| Asian | 1.2 | 3.5 | 2.2−5.4 | **<0.001** |
| Indigenous American* | 0.4 | 1.5 | 0.8−2.9 | 0.231 |
| BMI (kg/m²) | 0.0 | 1.0 | 0.9−1.0 | **0.044** |
| SLE duration | 0.0 | 1.0 | 1.0−1.0 | 0.377 |
| Extra-renal cSLEDAI-2K^‡^ | -0.1 | 0.9 | 0.9−0.9 | **<0.001** |
| SDI score | -0.4 | 0.6 | 0.5−0.8 | **<0.001** |
| Renal BILAG A−D | NA | NA | NA | NA |
| anti-dsDNA (+)^A^ | 0.3 | 1.4 | 0.9−2.1 | 0.126 |
| anti-Sm (+)^B^ | 0.5 | 1.7 | 1.1−2.6 | **0.022** |
| anti-Ribosomal P protein (+)^C^ | 0.6 | 1.8 | 1.2−2.9 | **0.009** |
| Any aCL(+) | 0.0 | 1.0 | 0.7−1.4 | 0.881 |
| Low C3^D^ | 0.8 | 2.2 | 1.6−3.1 | **<0.001** |
| Low C4 ^E^ | 0.2 | 1.3 | 0.9−1.7 | 0.119 |
| BAFF | 0.0 | 1.0 | 0.9−1.1 | 0.965 |
| Albumin (g/L) | -0.1 | 0.9 | 0.9−0.9 | **<0.001** |
| Proteinuria (g/24 h) | 0.3 | 1.4 | 1.3−1.5 | **<0.001** |
| Creatinine (µmol/L) | 0.0 | 1.0 | 1.0−1.0 | 0.943 |
| Treatment at baseline |  |  |  |  |
| Prednisone (or equivalent) average dose | 0.0 | 1.0 | 1.0−1.0 | 0.128 |
| Glucocorticoid use | 0.6 | 1.8 | 0.8−3.8 | 0.129 |
| AMA† | -0.3 | 0.7 | 0.5−1.0 | **0.022** |
| Azathioprine | -0.1 | 0.9 | 0.6−1.3 | 0.491 |
| Mycofenolate mofetil or sodium | 0.4 | 1.6 | 1.1−2.1 | **0.006** |
| Metothrexate | 0.0 | 1.0 | 0.6−1.7 | 0.980 |
| Trial intervention |  |  |  |  |
| Belimumab |  |  |  |  |
| i.v. 1 mg/kg (every 4^th^ week) | -1.0 | 0.4 | 0.2−0.7 | **0.001** |
| i.v. 10 mg/kg (every 4^th^ week) | -0.2 | 0.8 | 0.6−1.1 | 0.141 |
| s.c. 200 mg (weekly) | -0.5 | 0.6 | 0.4−1.0 | **0.030** |

Results from univariable proportional hazards (Cox) regression analysis, with data from the pooled BLISS study population. Proteinuria was estimated by 24-hour urine protein excretion, or the urine protein to creatinine ratio (UPCR). Data are presented as the coefficients, hazard ratios (HR), 95% confidence intervals (CI), and P values. Statistically significant P values are in bold. BMI: body mass index; SLE: systemic lupus erythematosus; cSLEDAI-2K: clinical Systemic Lupus Erythematous Disease Activity Index 2000; SDI: SLICC/ACR damage index; BILAG: British Isles Lupus Assessment Group; Sm: Smith; aCL: anticardiolipin antibody; C3: complement component 3; BAFF: B cell activating factor belonging to the TNF family; AMA: antimalarial agents; i.v.: intravenous; s.c.: subcutaneous NA: Not applicable.

*Alaska Native or American Indian from North, South or Central America.

^†^ Hydroxychloroquine, chloroquine, mepacrine, mepacrine hydrochloride or quinine sulfate.

^‡^SLEDAI-2K after suppression of serological (anti-dsDNA, C3 and C4) and renal descriptors (proteinuria, haematuria, pyuria and urinary casts).

**Cut-off for antibody positivity:**

^A^anti-dsDNA ≥30 IU/mL

^B^anti-Sm ≥15 U/mL

^C^anti-ribosomal P protein >25 EU/mL

**Cut-off for low complement levels:**

^D^C3 <90 mg/dL

^E^C4 <16 mg/dL in BLISS-52 and BLISS-76 and <10 mg/dL in BLISS-SC and BLISS-NEA

**Supplementary Table S6.** Hazard for renal flare development in patients with no prior renal involvement (renal BILAG E).

|  | Coefficient | HR | 95% CI | P value |
| --- | --- | --- | --- | --- |
| Patient characteristics | | | | |
| Age (years) | 0.0 | 1.0 | 0.9−1.0 | 0.637 |
| Male sex | NA | NA | NA | NA |
| Ethnicity (ref.: White/Caucasian) |  |  |  |  |
| African American | NA | NA | NA | NA |
| Asian | NA | NA | NA | NA |
| Indigenous American* | NA | NA | NA | NA |
| BMI (kg/m²) | 0.0 | 1.0 | 0.9−1.1 | 0.400 |
| SLE duration | 0.0 | 1.0 | 0.9−1.1 | 0.801 |
| Extra-renal cSLEDAI-2K^‡^ | 0.0 | 1.0 | 0.8−1.2 | 0.816 |
| SDI score | -0.4 | 0.7 | 0.3−1.5 | 0.322 |
| Renal BILAG A−D | NA | NA | NA | NA |
| anti-dsDNA (+)^A^ | 0.5 | 1.7 | 0.5−6.5 | 0.423 |
| anti-Sm (+)^B^ | 0.1 | 1.2 | 0.2−5.8 | 0.856 |
| anti-Ribosomal P protein (+)^C^ | 0.0 | 1.0 | 0.1−8.2 | 0.994 |
| Any aCL(+) | 0.0 | 1.0 | 0.2−4.6 | 0.983 |
| Low C3^D^ | 0.7 | 2.0 | 0.6−6.4 | 0.270 |
| Low C4^E^ | -0.7 | 0.5 | 0.1−1.9 | 0.325 |
| BAFF | 0.0 | 1.0 | 0.5−1.7 | 0.882 |
| Albumin (g/L) | 0.0 | 1.0 | 0.8−1.0 | 0.169 |
| Proteinuria (g/24 h) | 0.6 | 1.8 | 0.1−21.2 | 0.656 |
| Creatinine (µmol/L) | 0.0 | 1.0 | 1.0−1.0 | 0.983 |
| Treatment at baseline |  |  |  |  |
| Prednisone (or equivalent) average dose | 0.0 | 1.0 | 0.9−1.1 | 0.963 |
| Glucocorticoid use | -0.5 | 0.6 | 0.2−2.3 | 0.450 |
| AMA† | -0.9 | 0.4 | 0.1−1.4 | 0.147 |
| Azathioprine | 0.0 | 1.0 | 0.2−4.5 | 0.980 |
| Mycofenolate mofetil or sodium | 0.2 | 1.2 | 0.1−9.1 | 0.886 |
| Metothrexate | -0.7 | 0.5 | 0.1−4.0 | 0.528 |
| Trial intervention |  |  |  |  |
| Belimumab |  |  |  |  |
| i.v. 1 mg/kg (every 4^th^ week) | -1.4 | 0.3 | 0.0−2.2 | 0.209 |
| i.v. 10 mg/kg (every 4^th^ week) | -0.8 | 0.4 | 0.1−2.2 | 0.320 |
| s.c. 200 mg (weekly) | 0.3 | 1.3 | 0.3−5.6 | 0.739 |

Results from univariable proportional hazards (Cox) regression analysis, with data from the pooled BLISS study population. Proteinuria was estimated by 24-hour urine protein excretion, or the urine protein to creatinine ratio (UPCR). Data are presented as the coefficients, hazard ratios (HR), 95% confidence intervals (CI), and P values. Statistically significant P values are in bold. BMI: body mass index; SLE: systemic lupus erythematosus; cSLEDAI-2K: clinical Systemic Lupus Erythematous Disease Activity Index 2000; SDI: SLICC/ACR damage index; BILAG: British Isles Lupus Assessment Group; Sm: Smith; aCL: anticardiolipin antibody; C3: complement component 3; BAFF: B cell activating factor belonging to the TNF family; AMA: antimalarial agents; i.v.: intravenous; s.c.: subcutaneous; NA: Not applicable.

*Alaska Native or American Indian from North, South or Central America.

^†^ Hydroxychloroquine, chloroquine, mepacrine, mepacrine hydrochloride or quinine sulfate.

^‡^SLEDAI-2K after suppression of serological (anti-dsDNA, C3 and C4) and renal descriptors (proteinuria, haematuria, pyuria and urinary casts).

**Cut-off for antibody positivity:**

^A^anti-dsDNA ≥30 IU/mL

^B^anti-Sm ≥15 U/mL

^C^anti-ribosomal P protein >25 EU/mL

**Cut-off for low complement levels:**

^D^C3 <90 mg/dL

^E^C4 <16 mg/dL in BLISS-52 and BLISS-76 and <10 mg/dL in BLISS-SC and BLISS-NEA

**Supplementary Table S7.** Hazard for renal flare development in patients who received belimumab.

|  | Coefficient | HR | 95% CI | P value |
| --- | --- | --- | --- | --- |
| Patient characteristics | | | | |
| Age (years) | 0.0 | 1.0 | 0.9−1.0 | **<0.001** |
| Male sex | 0.4 | 1.5 | 0.7−2.9 | 0.285 |
| Ethnicity (ref.: White/Caucasian) |  |  |  |  |
| African American | 0.7 | 2.1 | 0.8−5.3 | 0.114 |
| Asian | 1.6 | 5.1 | 3.0−8.6 | **<0.001** |
| Indigenous American* | 0.3 | 1.4 | 0.6−3.2 | 0.458 |
| BMI (kg/m²) | -0.1 | 0.9 | 0.9−1.0 | **0.006** |
| SLE duration | 0.0 | 1.0 | 1.0−1.0 | 0.360 |
| Extra-renal cSLEDAI-2K^‡^ | -0.1 | 0.9 | 0.8−0.9 | **<0.001** |
| SDI score | -0.5 | 0.6 | 0.5−0.8 | **0.002** |
| Renal BILAG A−D | 2.8 | 15.9 | 7.0−36.3 | **<0.001** |
| anti-dsDNA (+)^A^ | 0.8 | 2.3 | 1.3−4.0 | **0.002** |
| anti-Sm (+)^B^ | 0.3 | 1.4 | 0.8−2.5 | 0.275 |
| anti-Ribosomal P protein (+)^C^ | 1.0 | 2.8 | 1.5−5.0 | **0.001** |
| Any aCL(+) | 0.4 | 1.5 | 0.9−2.4 | 0.084 |
| Low C3^D^ | 1.2 | 3.2 | 2.1−5.0 | **<0.001** |
| Low C4 ^E^ | 0.3 | 1.3 | 0.9−1.9 | 0.157 |
| BAFF | 0.40 | 1.0 | 0.9−1.1 | 0.725 |
| Albumin (g/L) | -0.2 | 0.8 | 0.8−0.9 | **<0.001** |
| Proteinuria (g/24 h) | 0.5 | 1.6 | 1.5−1.8 | **<0.001** |
| Creatinine (µmol/L) | 0.0 | 1.0 | 1.0−1.0 | 0.123 |
| Treatment at baseline |  |  |  |  |
| Prednisone (or equivalent) average dose | 0.0 | 1.0 | 1.0−1.0 | **0.002** |
| Glucocorticoid use | 1.5 | 4.6 | 1.5−14.5 | **0.009** |
| AMA† | -0.5 | 0.6 | 0.4−0.9 | **0.007** |
| Azathioprine | -0.5 | 0.6 | 0.3−1.1 | 0.076 |
| Mycofenolate mofetil or sodium | 0.9 | 2.4 | 1.6−3.6 | **<0.001** |
| Metothrexate | -0.4 | 0.7 | 0.3−1.4 | 0.290 |

Results from univariable proportional hazards (Cox) regression analysis, with data from the pooled BLISS study population. Proteinuria was estimated by 24-hour urine protein excretion, or the urine protein to creatinine ratio (UPCR). Data are presented as the coefficients, hazard ratios (HR), 95% confidence intervals (CI), and P values. Statistically significant P values are in bold. BMI: body mass index; SLE: systemic lupus erythematosus; cSLEDAI-2K: clinical Systemic Lupus Erythematous Disease Activity Index 2000; SDI: SLICC/ACR damage index; BILAG: British Isles Lupus Assessment Group; Sm: Smith; aCL: anticardiolipin antibody; C3: complement component 3; BAFF: B cell activating factor belonging to the TNF family; AMA: antimalarial agents.

*Alaska Native or American Indian from North, South or Central America.

^†^ Hydroxychloroquine, chloroquine, mepacrine, mepacrine hydrochloride or quinine sulfate.

^‡^SLEDAI-2K after suppression of serological (anti-dsDNA, C3 and C4) and renal descriptors (proteinuria, haematuria, pyuria and urinary casts).

**Cut-off for antibody positivity:**

^A^anti-dsDNA ≥30 IU/mL

^B^anti-Sm ≥15 U/mL

^C^anti-ribosomal P protein >25 EU/mL

**Cut-off for low complement levels:**

^D^C3 <90 mg/dL

^E^C4 <16 mg/dL in BLISS-52 and BLISS-76 and <10 mg/dL in BLISS-SC and BLISS-NEA

**Supplementary Table S8.** Hazard for renal flare development in patients with current or prior renal involvement (renal BILAG A−D) who received belimumab.

|  | Coefficient | HR | 95% CI | P value |
| --- | --- | --- | --- | --- |
| Patient characteristics | | | | |
| Age (years) | 0.0 | 1.0 | 0.9−1.0 | **<0.001** |
| Male sex | 0.2 | 1.2 | 0.6−2.4 | 0.555 |
| Ethnicity (ref.: White/Caucasian) |  |  |  |  |
| African American | 0.7 | 2.0 | 0.8−5.3 | 0.149 |
| Asian | 1.1 | 3.0 | 1.7−5.4 | **<0.001** |
| Indigenous American* | 0.3 | 1.3 | 0.5−3.1 | 0.571 |
| BMI (kg/m²) | 0.0 | 1.0 | 0.9−1.0 | 0.167 |
| SLE duration | 0.0 | 1.0 | 0.9−1.0 | 0.199 |
| Extra-renal cSLEDAI-2K^‡^ | -0.1 | 0.9 | 0.9−1.0 | **0.013** |
| SDI score | -0.4 | 0.7 | 0.5−0.9 | **0.007** |
| Renal BILAG A−D | NA | NA | NA | NA |
| anti-dsDNA (+)^A^ | 0.4 | 1.5 | 0.8−2.7 | 0.178 |
| anti-Sm (+)^B^ | 0.0 | 1.0 | 0.5−1.8 | 0.959 |
| anti-Ribosomal P protein (+)^C^ | 0.8 | 2.3 | 1.3−2.2 | **0.007** |
| Any aCL(+) | 0.5 | 1.6 | 1.0−2.5 | 0.053 |
| Low C3^D^ | 0.9 | 2.4 | 1.5−3.9 | **<0.001** |
| Low C4 ^E^ | 0.3 | 1.4 | 1.0−2.1 | 0.084 |
| BAFF | 0.0 | 1.0 | 0.9−1.1 | 0.721 |
| Albumin (g/L) | -0.1 | 0.9 | 0.8−0.9 | **<0.001** |
| Proteinuria (g/24 h) | 0.3 | 1.4 | 1.3−1.6 | **<0.001** |
| Creatinine (µmol/L) | 0.0 | 1.0 | 1.0−1.0 | 0.215 |
| Treatment at baseline |  |  |  |  |
| Prednisone (or equivalent) average dose | 0.0 | 1.0 | 1.0−1.0 | 0.277 |
| Glucocorticoid use | 1.3 | 3.6 | 0.9−14.7 | 0.071 |
| AMA† | -0.6 | 0.6 | 0.4−0.8 | **0.004** |
| Azathioprine | -0.5 | 0.6 | 0.4−1.1 | 0.101 |
| Mycofenolate mofetil or sodium | 0.5 | 1.6 | 1.1−2.4 | **0.023** |
| Metothrexate | 0.1 | 1.1 | 0.5−2.2 | 0.857 |

Results from univariable proportional hazards (Cox) regression analysis, with data from the pooled BLISS study population. Proteinuria was estimated by 24-hour urine protein excretion, or the urine protein to creatinine ratio (UPCR). Data are presented as the coefficients, hazard ratios (HR), 95% confidence intervals (CI), and P values. Statistically significant P values are in bold. BMI: body mass index; SLE: systemic lupus erythematosus; cSLEDAI-2K: clinical Systemic Lupus Erythematous Disease Activity Index 2000; SDI: SLICC/ACR damage index; BILAG: British Isles Lupus Assessment Group; Sm: Smith; aCL: anticardiolipin antibody; C3: complement component 3; BAFF: B cell activating factor belonging to the TNF family; AMA: antimalarial agents.

*Alaska Native or American Indian from North, South or Central America.

^†^ Hydroxychloroquine, chloroquine, mepacrine, mepacrine hydrochloride or quinine sulfate.

^‡^SLEDAI-2K after suppression of serological (anti-dsDNA, C3 and C4) and renal descriptors (proteinuria, haematuria, pyuria and urinary casts).

**Cut-off for antibody positivity:**

^A^anti-dsDNA ≥30 IU/mL

^B^anti-Sm ≥15 U/mL

^C^anti-ribosomal P protein >25 EU/mL

**Cut-off for low complement levels:**

^D^C3 <90 mg/dL

^E^C4 <16 mg/dL in BLISS-52 and BLISS-76 and <10 mg/dL in BLISS-SC and BLISS-NEA

**Supplementary Table S9.** Hazard for renal flare development in the placebo group.

|  | Coefficient | HR | 95% CI | P value |
| --- | --- | --- | --- | --- |
| Patient characteristics | | | | |
| Age (years) | 0.0 | 1.0 | 1.0−1.0 | **0.008** |
| Male sex | 0.4 | 1.5 | 0.7−3.1 | 0.261 |
| Ethnicity (ref.: White/Caucasian) |  |  |  |  |
| African American | 0.4 | 1.5 | 0.4−5.4 | 0.535 |
| Asian | 2.0 | 7.8 | 4.0−15.1 | **<0.001** |
| Indigenous American* | 0.0 | 1.5 | 1.0−6.2 | 0.051 |
| BMI (kg/m²) | -0.1 | 0.9 | 0.9−1.0 | **0.011** |
| SLE duration | 0.0 | 1.0 | 1.0−1.0 | 0.963 |
| Extra-renal cSLEDAI-2K^‡^ | -0.2 | 0.8 | 0.8−0.9 | **<0.001** |
| SDI score | -0.5 | 0.6 | 0.4−0.8 | **0.002** |
| Renal BILAG A−D | 5.8 | 15.6 | 5.9−36.1 | **<0.001** |
| anti-dsDNA (+)^A^ | 0.7 | 2.0 | 1.1−3.5 | **0.014** |
| anti-Sm (+)^B^ | 1.3 | 3.7 | 2.0−6.9 | **<0.001** |
| anti-Ribosomal P protein (+)^C^ | 0.6 | 1.7 | 0.9−3.4 | 0.106 |
| Any aCL(+) | -0.9 | 0.4 | 0.2−0.9 | **0.021** |
| Low C3^D^ | 1.0 | 2.7 | 1.7−4.4 | **<0.001** |
| Low C4 ^E^ | 0.2 | 1.2 | 0.8−1.8 | 0.492 |
| BAFF | 0.0 | 1.0 | 0.8−1.2 | 0.958 |
| Albumin (g/L) | -0.2 | 0.9 | 0.8−0.9 | **<0.001** |
| Proteinuria (g/24 h) | 0.4 | 1.5 | 1.4−1.7 | **<0.001** |
| Creatinine (µmol/L) | 0.0 | 1.0 | 1.0−1.0 | 0.325 |
| Treatment at baseline |  |  |  |  |
| Prednisone (or equivalent) average dose | 0.0 | 1.0 | 1.0−1.0 | **0.036** |
| Glucocorticoid use | 0.4 | 1.5 | 0.7−3.7 | 0.271 |
| AMA† | -0.1 | 0.9 | 0.6−1.5 | 0.715 |
| Azathioprine | 0.3 | 1.4 | 0.8−2.3 | 0.204 |
| Mycofenolate mofetil or sodium | 0.8 | 2.3 | 1.4−3.7 | **<0.001** |
| Metothrexate | -0.4 | 0.6 | 0.3−1.3 | 0.241 |

Results from univariable proportional hazards (Cox) regression analysis, with data from the pooled BLISS study population. Proteinuria was estimated by 24-hour urine protein excretion, or the urine protein to creatinine ratio (UPCR). Data are presented as the coefficients, hazard ratios (HR), 95% confidence intervals (CI), and P values. Statistically significant P values are in bold. BMI: body mass index; SLE: systemic lupus erythematosus; cSLEDAI-2K: clinical Systemic Lupus Erythematous Disease Activity Index 2000; SDI: SLICC/ACR damage index; BILAG: British Isles Lupus Assessment Group; Sm: Smith; aCL: anticardiolipin antibody; C3: complement component 3; BAFF: B cell activating factor belonging to the TNF family; AMA: antimalarial agents.

*Alaska Native or American Indian from North, South or Central America.

^†^ Hydroxychloroquine, chloroquine, mepacrine, mepacrine hydrochloride or quinine sulfate.

^‡^SLEDAI-2K after suppression of serological (anti-dsDNA, C3 and C4) and renal descriptors (proteinuria, haematuria, pyuria and urinary casts).

**Cut-off for antibody positivity:**

^A^anti-dsDNA ≥30 IU/mL

^B^anti-Sm ≥15 U/mL

^C^anti-ribosomal P protein >25 EU/mL

**Cut-off for low complement levels:**

^D^C3 <90 mg/dL

^E^C4 <16 mg/dL in BLISS-52 and BLISS-76 and <10 mg/dL in BLISS-SC and BLISS-NEA

**Supplementary Table S10.** Hazard for renal flare development in patients with current or prior renal involvement (renal BILAG A−D) who received placebo.

|  | Coefficient | HR | 95% CI | P value |
| --- | --- | --- | --- | --- |
| Patient characteristics | | | | |
| Age (years) | 0.0 | 1.0 | 0.9−1.0 | 0.053 |
| Male sex | 0.3 | 1.4 | 0.7−2.8 | 0.404 |
| Ethnicity (ref.: White/Caucasian) |  |  |  |  |
| African American | 0.4 | 1.5 | 0.4−5.4 | 0.571 |
| Asian | 1.4 | 4.2 | 2.1−8.6 | **<0.001** |
| Indigenous American* | 0.7 | 2.0 | 0.7−5.6 | 0.187 |
| BMI (kg/m²) | 0.0 | 1.0 | 0.9−1.0 | 0.113 |
| SLE duration | 0.0 | 1.0 | 1.0−1.0 | 0.865 |
| Extra-renal cSLEDAI-2K^‡^ | -0.1 | 0.9 | 0.8−0.9 | **<0.001** |
| SDI score | -0.5 | 0.6 | 0.4−0.9 | **0.010** |
| Renal BILAG A−D | NA | NA | NA | NA |
| anti-dsDNA (+)^A^ | 0.3 | 1.4 | 0.8−2.4 | 0.294 |
| anti-Sm (+)^B^ | 1.2 | 3.2 | 1.6−6.3 | **<0.001** |
| anti-Ribosomal P protein (+)^C^ | 0.3 | 1.3 | 0.7−2.7 | 0.444 |
| Any aCL(+) | -1.0 | 0.4 | 0.2−0.9 | **0.025** |
| Low C3^D^ | 0.7 | 2.0 | 1.3−3.3 | **0.004** |
| Low C4 ^E^ | 0.1 | 1.1 | 0.7−1.7 | 0.658 |
| BAFF | 0.0 | 1.0 | 0.8−1.1 | 0.562 |
| Albumin (g/L) | -0.1 | 0.9 | 0.9−0.9 | **<0.001** |
| Proteinuria (g/24 h) | 0.3 | 1.3 | 1.2−1.5 | **<0.001** |
| Creatinine (µmol/L) | 0.0 | 1.0 | 1.0−1.0 | 0.160 |
| Treatment at baseline |  |  |  |  |
| Prednisone (or equivalent) average dose | 0.0 | 1.0 | 1.0−1.0 | 0.252 |
| Glucocorticoid use | 0.1 | 1.1 | 0.4−2.7 | 0.851 |
| AMA† | 0.0 | 1.0 | 0.6−1.6 | 0.914 |
| Azathioprine | 0.3 | 1.4 | 0.8−2.3 | 0.266 |
| Mycofenolate mofetil or sodium | 0.4 | 1.5 | 0.9−2.4 | 0.109 |
| Metothrexate | -0.1 | 0.9 | 0.4−1.9 | 0.735 |

Results from univariable proportional hazards (Cox) regression analysis, with data from the pooled BLISS study population. Proteinuria was estimated by 24-hour urine protein excretion, or the urine protein to creatinine ratio (UPCR). Data are presented as the coefficients, hazard ratios (HR), 95% confidence intervals (CI), and P values. Statistically significant P values are in bold. BMI: body mass index; SLE: systemic lupus erythematosus; cSLEDAI-2K: clinical Systemic Lupus Erythematous Disease Activity Index 2000; SDI: SLICC/ACR damage index; BILAG: British Isles Lupus Assessment Group; Sm: Smith; aCL: anticardiolipin antibody; C3: complement component 3; BAFF: B cell activating factor belonging to the TNF family; AMA: antimalarial agents.

*Alaska Native or American Indian from North, South or Central America.

^†^ Hydroxychloroquine, chloroquine, mepacrine, mepacrine hydrochloride or quinine sulfate.

^‡^SLEDAI-2K after suppression of serological (anti-dsDNA, C3 and C4) and renal descriptors (proteinuria, haematuria, pyuria and urinary casts).

**Cut-off for antibody positivity:**

^A^anti-dsDNA ≥30 IU/mL

^B^anti-Sm ≥15 U/mL

^C^anti-ribosomal P protein >25 EU/mL

**Cut-off for low complement levels:**

^D^C3 <90 mg/dL

^E^C4 <16 mg/dL in BLISS-52 and BLISS-76 and <10 mg/dL in BLISS-SC and BLISS-NEA

**Supplementary Table S11.** Adjusted hazard for renal flare development in the entire study.

|  | **Coefficient** | **HR** | **95% CI** | **P value** |
| --- | --- | --- | --- | --- |
| **Multivariable model including demographics and clinical characteristic as covariates** | | | | |
| Age (years) | 0.0 | 1.0 | 1.0−1.0 | 0.058 |
| Male sex | 0.2 | 1.2 | 0.7−2.0 | 0.503 |
| Ethnicity (ref.: White/Caucasian) |  |  |  |  |
| African American | 0.4 | 1.6 | 0.7−3.4 | 0.254 |
| Asian | 0.9 | 2.6 | 1.6−4.0 | **<0.001** |
| Indigenous American* | 0.3 | 1.4 | 0.7−2.5 | 0.337 |
| BMI (kg/m²) | 0.0 | 1.0 | 1.0−1.0 | 0.721 |
| Extra-renal cSLEDAI-2K^‡^ | -0.1 | 0.9 | 0.9−1.0 | **0.020** |
| SDI score | -0.3 | 0.7 | 0.6−0.9 | **0.008** |
| Renal BILAG A−D | 2.2 | 9.4 | 5.0−17.7 | **<0.001** |
| Treatment at baseline |  |  |  |  |
| Prednisone (or equivalent) average dose | 0.0 | 1.0 | 1.0−1.0 | 0.820 |
| AMA† | -0.5 | 0.6 | 0.5−0.8 | **0.001** |
| Azathioprine | 0.0 | 1.0 | 0.7−1.6 | 0.845 |
| Mycofenolate mofetil or sodium | 0.3 | 1.3 | 0.9−1.8 | 0.142 |
| Metothrexate | 0.1 | 1.2 | 0.7−2.1 | 0.424 |
| Trial intervention |  |  |  |  |
| Belimumab use (any dose) | -0.5 | 0.6 | 0.5−0.8 | **0.002** |
| **Results from multivariable models, one for each candidate predictor, all adjusting for age, sex, ethnicity, BMI, extra-renal disease activity, organ damage, nephritis history, and use of prednisone, antimalarials, immunosuppressants, and belimumab** | | | | |
| anti-dsDNA (+)^A^ | 0.2 | 1.2 | 0.8−1.8 | 0.350 |
| anti-Sm (+)^B^ | 0.3 | 1.4 | 0.9−2.2 | 0.114 |
| anti-Ribosomal P protein (+)^C^ | 0.5 | 1.7 | 1.0−2.6 | **0.034** |
| Low C3^D^ | 0.6 | 1.8 | 1.3−2.5 | **<0.001** |
| Low C4^E^ | 0.3 | 1.3 | 1.0−1.8 | 0.060 |
| BAFF | 0,0 | 1.0 | 1.0−1.1 | 0.246 |
| Any aCL (+) | 0.0 | 1.0 | 0.7−1.5 | 0.869 |
| Creatinine (µmol/L) | 0.0 | 1.0 | 1.0−1.0 | 0.151 |
| Proteinuria (g/24 h) | 0.3 | 1.3 | 1.2−1.4 | **<0.001** |
| Albumin (g/L) | -0.1 | 0.9 | 0.9−0.9 | **<0.001** |

Results from multivariable proportional hazards (Cox) regression analysis, with data from the pooled BLISS study population. Proteinuria was estimated by 24-hour urine protein excretion, or the urine protein to creatinine ratio (UPCR). Data are presented as the coefficients, hazard ratios (HR), 95% confidence intervals (CI), and P values. Statistically significant P values are in bold. BMI: body mass index; cSLEDAI-2K: clinical Systemic Lupus Erythematous Disease Activity Index 2000; SDI: SLICC/ACR damage index; BILAG: British Isles Lupus Assessment Group; AMA: antimalarial agents; Sm: Smith; C3: complement component 3; C4: complement component 4; BAFF: B cell activating factor belonging to the TNF family; aCL: anticardiolipin antibody.

*Alaska Native or American Indian from North, South or Central America.

^†^ Hydroxychloroquine, chloroquine, mepacrine, mepacrine hydrochloride or quinine sulfate.

^‡^SLEDAI-2K after suppression of serological (anti-dsDNA, C3 and C4) and renal descriptors (proteinuria, haematuria, pyuria and urinary casts).

**Cut-off for antibody positivity:**

^A^anti-dsDNA ≥30 IU/mL

^B^anti-Sm ≥15 U/mL

^C^anti-ribosomal P protein >25 EU/mL

**Cut-off for low complement levels:**

^D^C3 <90 mg/dL

^E^C4 <16 mg/dL in BLISS-52 and BLISS-76 and <10 mg/dL in BLISS-SC and BLISS-NEA

**Supplementary Table S12.** Adjusted hazard for renal flare development in patients with current or prior renal involvement (renal BILAG A−D).

|  | **Coefficient** | **HR** | **95% CI** | **P value** |
| --- | --- | --- | --- | --- |
| **Multivariable model including demographics and clinical characteristic as covariates** | | | | |
| Age (years) | 0.0 | 1.0 | 1.0−1.0 | 0.057 |
| Male sex | 0.2 | 1.2 | 0.7−2.1 | 0.413 |
| Ethnicity (ref.: White/Caucasian) |  |  |  |  |
| African American | 0.6 | 1.8 | 0.8−4.0 | 0.140 |
| Asian | 1.0 | 2.7 | 1.7−4.3 | **<0.001** |
| Indigenous American* | 0.3 | 1.4 | 0.7−2.7 | 0.339 |
| BMI (kg/m²) | 0.0 | 1.0 | 1.0−1.0 | 0.666 |
| Extra-renal cSLEDAI-2K^‡^ | -0.1 | 0.9 | 0.9−1.0 | **0.014** |
| SDI score | -0.3 | 0.7 | 0.6−1.0 | **0.012** |
| Renal BILAG A−D | NA | NA | NA | NA |
| Treatment at baseline |  |  |  |  |
| Prednisone (or equivalent) average dose | 0.0 | 1.0 | 1.0−1.0 | 0.858 |
| AMA† | -0.5 | 0.6 | 0.5−0.9 | **0.003** |
| Azathioprine | 0.1 | 1.1 | 0.7−1.6 | 0.781 |
| Mycofenolate mofetil or sodium | 0.2 | 1.3 | 0.9−1.8 | 0.160 |
| Metothrexate | 0.3 | 1.3 | 0.8−2.3 | 0.293 |
| Trial intervention |  |  |  |  |
| Belimumab use (any dose) | -0.5 | 0.6 | 0.5−0.9 | **0.003** |
| **Results from multivariable models, one for each candidate predictor, all adjusting for age, sex, ethnicity, BMI, extra-renal disease activity, organ damage, nephritis history, and use of prednisone, antimalarials, immunosuppressants, and belimumab** | | | | |
| anti-dsDNA (+)^A^ | 0.2 | 1.2 | 0.8−1.8 | 0.414 |
| anti-Sm (+)^B^ | 0.4 | 1.4 | 0.9−2.2 | 0.126 |
| anti-Ribosomal P protein (+)^C^ | 0.5 | 1.7 | 1.1−2.8 | **0.029** |
| Low C3^D^ | 0.6 | 1.8 | 1.3−2.5 | **0.001** |
| Low C4^E^ | 0.4 | 1.4 | 1.1−2.0 | 0.021 |
| BAFF | 0.0 | 1.0 | 1.0−1.1 | 0.215 |
| Any aCL (+) | 0.1 | 1.1 | 0.7−1.6 | 0.809 |
| Creatinine (µmol/L) | 0.0 | 1.0 | 1.0−1.0 | 0.180 |
| Proteinuria (g/24 h) | 0.3 | 1.3 | 1.2−1.4 | **<0.001** |
| Albumin (g/L) | -0.1 | 0.9 | 0.9−0.9 | **<0.001** |

Results from multivariable proportional hazards (Cox) regression analysis, with data from the pooled BLISS study population. Proteinuria was estimated by 24-hour urine protein excretion, or the urine protein to creatinine ratio (UPCR). Data are presented as the coefficients, hazard ratios (HR), 95% confidence intervals (CI), and P values. Statistically significant P values are in bold. BMI: body mass index; cSLEDAI-2K: clinical Systemic Lupus Erythematous Disease Activity Index 2000; SDI: SLICC/ACR damage index; BILAG: British Isles Lupus Assessment Group; AMA: antimalarial agents; Sm: Smith; C3: complement component 3; C4: complement component 4; BAFF: B cell activating factor belonging to the TNF family; aCL: anticardiolipin antibody.

*Alaska Native or American Indian from North, South or Central America.

^†^ Hydroxychloroquine, chloroquine, mepacrine, mepacrine hydrochloride or quinine sulfate.

^‡^SLEDAI-2K after suppression of serological (anti-dsDNA, C3 and C4) and renal descriptors (proteinuria, haematuria, pyuria and urinary casts).

**Cut-off for antibody positivity:**

^A^anti-dsDNA ≥30 IU/mL

^B^anti-Sm ≥15 U/mL

^C^anti-ribosomal P protein >25 EU/mL

**Cut-off for low complement levels:**

^D^C3 <90 mg/dL

^E^C4 <16 mg/dL in BLISS-52 and BLISS-76 and <10 mg/dL in BLISS-SC and BLISS-NEA

**Supplementary Table S13.** Adjusted hazard for renal flare development in patients who received belimumab.

|  | **Coefficient** | **HR** | **95% CI** | **P value** |
| --- | --- | --- | --- | --- |
| **Multivariable model including demographics and clinical characteristic as covariates** | | | | |
| Age (years) | 0.0 | 1.0 | 0.9−1.0 | **0.003** |
| Male sex | 0.2 | 1.2 | 0.6−2.4 | 0.573 |
| Ethnicity (ref.: White/Caucasian) |  |  |  |  |
| African American | 0.4 | 1.5 | 0.6−3.8 | 0.412 |
| Asian | 0.7 | 2.1 | 1.2−3.7 | **0.011** |
| Indigenous American* | 0.0 | 1.0 | 0.4−2.2 | 0.929 |
| BMI (kg/m²) | 0.0 | 1.0 | 1.0−1.1 | 0.540 |
| Extra-renal cSLEDAI-2K^‡^ | 0.0 | 1.0 | 0.9−1.0 | 0.371 |
| SDI score | -0.3 | 0.8 | 0.6−1.0 | 0.078 |
| Renal BILAG A−D | 2.4 | 10.6 | 4.5−24.7 | **<0.001** |
| Treatment at baseline |  |  |  |  |
| Prednisone (or equivalent) average dose | 0.0 | 1.0 | 1.0−1.0 | 0.796 |
| AMA† | -0.8 | 0.4 | 0.3−0.7 | **<0.001** |
| Azathioprine | -0.4 | 0.7 | 0.4−1.2 | 0.218 |
| Mycofenolate mofetil or sodium | 0.2 | 1.2 | 0.8−1.9 | 0.436 |
| Metothrexate | 0.2 | 1.2 | 0.6−2.5 | 0.643 |
| **Results from multivariable models, one for each candidate predictor, all adjusting for age, sex, ethnicity, BMI, extra-renal disease activity, organ damage, nephritis history, and use of prednisone, antimalarials, and immunosuppressants** | | | | |
| anti-dsDNA (+)^A^ | 0.2 | 1.2 | 0.7−2.1 | 0.567 |
| anti-Sm (+)^B^ | -0.3 | 0.8 | 0.4−1.4 | 0.408 |
| anti-Ribosomal P protein (+)^C^ | 0.6 | 1.8 | 1.0−3.4 | 0.053 |
| Low C3^D^ | 0.6 | 1.9 | 1.2−3.0 | **0.006** |
| Low C4^E^ | 0.3 | 1.4 | 0.9−2.1 | 0.089 |
| BAFF | 0.0 | 1.0 | 1.0−1.1 | 0.361 |
| Any aCL (+) | 0.6 | 1.8 | 1.1−2.8 | **0.020** |
| Creatinine (µmol/L) | 0.0 | 1.0 | 1.0−1.0 | **0.029** |
| Proteinuria (g/24 h) | 0.3 | 1.3 | 1.2−1.5 | **<0.001** |
| Albumin (g/L) | -0.1 | 0.9 | 0.9−0.9 | **<0.001** |

Results from multivariable proportional hazards (Cox) regression analysis, with data from the pooled BLISS study population. Proteinuria was estimated by 24-hour urine protein excretion, or the urine protein to creatinine ratio (UPCR). Data are presented as the coefficients, hazard ratios (HR), 95% confidence intervals (CI), and P values. Statistically significant P values are in bold. BMI: body mass index; cSLEDAI-2K: clinical Systemic Lupus Erythematous Disease Activity Index 2000; SDI: SLICC/ACR damage index; BILAG: British Isles Lupus Assessment Group; AMA: antimalarial agents; Sm: Smith; C3: complement component 3; C4: complement component 4; BAFF: B cell activating factor belonging to the TNF family; aCL: anticardiolipin antibody.

*Alaska Native or American Indian from North, South or Central America.

^†^ Hydroxychloroquine, chloroquine, mepacrine, mepacrine hydrochloride or quinine sulfate.

^‡^SLEDAI-2K after suppression of serological (anti-dsDNA, C3 and C4) and renal descriptors (proteinuria, haematuria, pyuria and urinary casts).

**Cut-off for antibody positivity:**

^A^anti-dsDNA ≥30 IU/mL

^B^anti-Sm ≥15 U/mL

^C^anti-ribosomal P protein >25 EU/mL

**Cut-off for low complement levels:**

^D^C3 <90 mg/dL

^E^C4 <16 mg/dL in BLISS-52 and BLISS-76 and <10 mg/dL in BLISS-SC and BLISS-NEA

**Supplementary Table S14.** Adjusted hazard for renal flare in patients with current or prior renal involvement (renal BILAG A−D) who received belimumab.

|  | **Coefficient** | **HR** | **95% CI** | **P value** |
| --- | --- | --- | --- | --- |
| **Multivariable model including demographics and clinical characteristic as covariates** | | | | |
| Age (years) | 0.0 | 1.0 | 0.9−1.0 | **0.005** |
| Male sex | 0.2 | 1.3 | 0.6−2.5 | 0.486 |
| Ethnicity (ref.: White/Caucasian) |  |  |  |  |
| African American | 0.6 | 1.8 | 0.7−4.7 | 0.252 |
| Asian | 0.9 | 2.4 | 1.3−4.4 | **0.006** |
| Indigenous American* | 0.1 | 1.2 | 0.5−2.8 | 0.750 |
| BMI (kg/m²) | 0.0 | 1.0 | 1.0−1.1 | 0.523 |
| Extra-renal cSLEDAI-2K^‡^ | 0.0 | 1.0 | 0.9−1.0 | 0.316 |
| SDI score | -0.3 | 0.8 | 0.6−1.0 | 0.098 |
| Renal BILAG A−D | NA | NA | NA | NA |
| Treatment at baseline |  |  |  |  |
| Prednisone (or equivalent) average dose | 0.0 | 1.0 | 1.0−1.0 | 0.691 |
| AMA† | -0.8 | 0.5 | 0.3−0.7 | **<0.001** |
| Azathioprine | -0.3 | 0.8 | 0.4−1.4 | 0.351 |
| Mycofenolate mofetil or sodium | 0.2 | 1.3 | 0.8−2.0 | 0.318 |
| Metothrexate | 0.3 | 1.4 | 0.7−3.0 | 0.389 |
| **Results from multivariable models, one for each candidate predictor, all adjusting for age, sex, ethnicity, BMI, extra-renal disease activity, organ damage, nephritis history, and use of prednisone, antimalarials, and immunosuppressants** | | | | |
| anti-dsDNA (+)^A^ | 0.2 | 1.2 | 0.6−2.2 | 0.541 |
| anti-Sm (+)^B^ | -0.3 | 0.8 | 0.4−1.4 | 0.379 |
| anti-Ribosomal P protein (+)^C^ | 0.8 | 2.2 | 1.1−4.2 | **0.017** |
| Low C3^D^ | 0.7 | 2.0 | 1.2−3.2 | **0.006** |
| Low C4^E^ | 0.4 | 1.6 | 1.0−2.4 | **0.032** |
| BAFF | 0.0 | 1.0 | 1.0−1.1 | 0.325 |
| Any aCL (+) | 0.6 | 1.8 | 1.1−3.0 | **0.014** |
| Creatinine (µmol/L) | 0.0 | 1.0 | 1.0−1.0 | **0.020** |
| Proteinuria (g/24 h) | 0.3 | 1.3 | 1.2−1.5 | **<0.001** |
| Albumin (g/L) | -0.1 | 0.9 | 0.9−0.9 | **<0.001** |

Results from multivariable proportional hazards (Cox) regression analysis, with data from the pooled BLISS study population. Proteinuria was estimated by 24-hour urine protein excretion, or the urine protein to creatinine ratio (UPCR). Data are presented as the coefficients, hazard ratios (HR), 95% confidence intervals (CI), and P values. Statistically significant P values are in bold. BMI: body mass index; cSLEDAI-2K: clinical Systemic Lupus Erythematous Disease Activity Index 2000; SDI: SLICC/ACR damage index; BILAG: British Isles Lupus Assessment Group; AMA: antimalarial agents; Sm: Smith; C3: complement component 3; C4: complement component 4; BAFF: B cell activating factor belonging to the TNF family; aCL: anticardiolipin antibody.

*Alaska Native or American Indian from North, South or Central America.

^†^ Hydroxychloroquine, chloroquine, mepacrine, mepacrine hydrochloride or quinine sulfate.

^‡^SLEDAI-2K after suppression of serological (anti-dsDNA, C3 and C4) and renal descriptors (proteinuria, haematuria, pyuria and urinary casts).

**Cut-off for antibody positivity:**

^A^anti-dsDNA ≥30 IU/mL

^B^anti-Sm ≥15 U/mL

^C^anti-ribosomal P protein >25 EU/mL

**Cut-off for low complement levels:**

^D^C3 <90 mg/dL

^E^C4 <16 mg/dL in BLISS-52 and BLISS-76 and <10 mg/dL in BLISS-SC and BLISS-NEA

**Supplementary Table S15.** Adjusted hazard for renal flare development in the placebo group.

|  | **Coefficient** | **HR** | **95% CI** | **P value** |
| --- | --- | --- | --- | --- |
| **Multivariable model including demographics and clinical characteristic as covariates** | | | | |
| Age (years) | 0.0 | 1.0 | 1.0−1.0 | 0.511 |
| Male sex | 0.3 | 1.3 | 0.6−2.8 | 0.482 |
| Ethnicity (ref.: White/Caucasian) |  |  |  |  |
| African American | 0.5 | 1.6 | 0.4−6.0 | 0.491 |
| Asian | 1.3 | 3.7 | 1.8−7.7 | **<0.001** |
| Indigenous American* | 0.9 | 2.4 | 0.9−6.2 | 0.067 |
| BMI (kg/m²) | 0.0 | 1.0 | 0.9−1.0 | 0.895 |
| Extra-renal cSLEDAI-2K^‡^ | -0.1 | 0.9 | 0.8−1.0 | **0.011** |
| SDI score | -0.4 | 0.7 | 0.5−1.0 | **0.029** |
| Renal BILAG A−D | 2.1 | 8.6 | 3.3−21.8 | **<0.001** |
| Treatment at baseline |  |  |  |  |
| Prednisone (or equivalent) average dose | 0.0 | 1.0 | 1.0−1.0 | 0.991 |
| AMA† | 0.0 | 1.0 | 0.6−1.5 | 0.850 |
| Azathioprine | 0.6 | 1.8 | 1.0−3.1 | **0.047** |
| Mycofenolate mofetil or sodium | 0.4 | 1.5 | 0.9−2.5 | 0.158 |
| Metothrexate | 0.3 | 1.3 | 0.6−2.8 | 0.496 |
| **Results from multivariable models, one for each candidate predictor, all adjusting for age, sex, ethnicity, BMI, extra-renal disease activity, organ damage, nephritis history, and use of prednisone, antimalarials, and immunosuppressants** | | | | |
| anti-dsDNA (+)^A^ | 0.2 | 1.3 | 0.7−2.3 | 0.397 |
| anti-Sm (+)^B^ | 1.1 | 2.9 | 1.5−5.6 | **0.002** |
| anti-Ribosomal P protein (+)^C^ | 0.2 | 1.2 | 0.6−2.5 | 0.575 |
| Low C3^D^ | 0.5 | 1.6 | 1.0−2.6 | 0.076 |
| Low C4^E^ | 0.2 | 1.2 | 0.8−2.0 | 0.350 |
| BAFF | 0.0 | 1.0 | 0.9−1.2 | 0.896 |
| Any aCL (+) | -0.9 | 0.4 | 0.2−0.9 | **0.028** |
| Creatinine (µmol/L) | 0.0 | 1.0 | 1.0−1.0 | 0.286 |
| Proteinuria (g/24 h) | 0.2 | 1.3 | 1.1−1.4 | **<0.001** |
| Albumin (g/L) | -0.1 | 0.9 | 0.9−0.9 | **<0.001** |

Results from multivariable proportional hazards (Cox) regression analysis, with data from the pooled BLISS study population. Proteinuria was estimated by 24-hour urine protein excretion, or the urine protein to creatinine ratio (UPCR). Data are presented as the coefficients, hazard ratios (HR), 95% confidence intervals (CI), and P values. Statistically significant P values are in bold. BMI: body mass index; cSLEDAI-2K: clinical Systemic Lupus Erythematous Disease Activity Index 2000; SDI: SLICC/ACR damage index; BILAG: British Isles Lupus Assessment Group; AMA: antimalarial agents; Sm: Smith; C3: complement component 3; C4: complement component 4; BAFF: B cell activating factor belonging to the TNF family; aCL: anticardiolipin antibody.

*Alaska Native or American Indian from North, South or Central America.

^†^ Hydroxychloroquine, chloroquine, mepacrine, mepacrine hydrochloride or quinine sulfate.

^‡^SLEDAI-2K after suppression of serological (anti-dsDNA, C3 and C4) and renal descriptors (proteinuria, haematuria, pyuria and urinary casts).

**Cut-off for antibody positivity:**

^A^anti-dsDNA ≥30 IU/mL

^B^anti-Sm ≥15 U/mL

^C^anti-ribosomal P protein >25 EU/mL

**Cut-off for low complement levels:**

^D^C3 <90 mg/dL

^E^C4 <16 mg/dL in BLISS-52 and BLISS-76 and <10 mg/dL in BLISS-SC and BLISS-NEA

**Supplementary Table S16.** Adjusted hazard for renal flare development in patients with current or prior renal involvement (renal BILAG A−D) who received placebo.

|  | **Coefficient** | **HR** | **95% CI** | **P value** |
| --- | --- | --- | --- | --- |
| **Multivariable model including demographics and clinical characteristic as covariates** | | | | |
| Age (years) | 0.0 | 1.0 | 1.0−1.0 | 0.712 |
| Male sex | 0.3 | 1.4 | 0.7−3.0 | 0.385 |
| Ethnicity (ref.: White/Caucasian) |  |  |  |  |
| African American | 0.6 | 0.9 | 0.5−7.2 | 0.379 |
| Asian | 1.3 | 3.6 | 1.6−7.7 | **0.001** |
| Indigenous American* | 0.7 | 2.0 | 0.7−5.9 | 0.195 |
| BMI (kg/m²) | 0.0 | 1.0 | 0.9−1.1 | 0.957 |
| Extra-renal cSLEDAI-2K^‡^ | -0.1 | 0.9 | 0.9−1.0 | **0.012** |
| SDI score | -0.4 | 0.7 | 0.5−1.0 | **0.044** |
| Renal BILAG A−D | NA | NA | NA | NA |
| Treatment at baseline |  |  |  |  |
| Prednisone (or equivalent) average dose | 0.0 | 1.0 | 1.0−1.0 | 0.847 |
| AMA† | 0.0 | 1.0 | 0.6−1.6 | 0.929 |
| Azathioprine | 0.5 | 1.7 | 0.9−3.1 | 0.080 |
| Mycofenolate mofetil or sodium | 0.3 | 1.3 | 0.8−2.3 | 0.305 |
| Metothrexate | 0.3 | 1.3 | 0.6−3.0 | 0.500 |
| **Results from multivariable models, one for each candidate predictor, all adjusting for age, sex, ethnicity, BMI, extra-renal disease activity, organ damage, nephritis history, and use of prednisone, antimalarials, and immunosuppressants** | | | | |
| anti-dsDNA (+)^A^ | 0.2 | 1.2 | 0.7−2.2 | 0.512 |
| anti-Sm (+)^B^ | 1.1 | 3.1 | 1.5−6.4 | **0.002** |
| anti-Ribosomal P protein (+)^C^ | 0.1 | 1.1 | 0.5−2.4 | 0.783 |
| Low C3^D^ | 0.5 | 1.6 | 0.9−2.7 | 0.088 |
| Low C4^E^ | 0.3 | 1.3 | 1.0−2.4 | 0.261 |
| BAFF | 0.0 | 1.0 | 0.9−1.2 | 0.794 |
| Any aCL (+) | -1.0 | 0.4 | 0.2−0.9 | **0.026** |
| Creatinine (µmol/L) | 0.0 | 1.0 | 1.0−1.0 | 0.198 |
| Proteinuria (g/24 h) | 0.2 | 1.3 | 1.1−1.4 | **<0.001** |
| Albumin (g/L) | -0.1 | 0.9 | 0.9−0.9 | **<0.001** |

Results from multivariable proportional hazards (Cox) regression analysis, with data from the pooled BLISS study population. Proteinuria was estimated by 24-hour urine protein excretion, or the urine protein to creatinine ratio (UPCR). Data are presented as the coefficients, hazard ratios (HR), 95% confidence intervals (CI), and P values. Statistically significant P values are in bold. BMI: body mass index; cSLEDAI-2K: clinical Systemic Lupus Erythematous Disease Activity Index 2000; SDI: SLICC/ACR damage index; BILAG: British Isles Lupus Assessment Group; AMA: antimalarial agents; Sm: Smith; C3: complement component 3; C4: complement component 4; BAFF: B cell activating factor belonging to the TNF family; aCL: anticardiolipin antibody.

*Alaska Native or American Indian from North, South or Central America.

^†^ Hydroxychloroquine, chloroquine, mepacrine, mepacrine hydrochloride or quinine sulfate.

^‡^SLEDAI-2K after suppression of serological (anti-dsDNA, C3 and C4) and renal descriptors (proteinuria, haematuria, pyuria and urinary casts).

**Cut-off for antibody positivity:**

^A^anti-dsDNA ≥30 IU/mL

^B^anti-Sm ≥15 U/mL

^C^anti-ribosomal P protein >25 EU/mL

**Cut-off for low complement levels:**

^D^C3 <90 mg/dL

^E^C4 <16 mg/dL in BLISS-52 and BLISS-76 and <10 mg/dL in BLISS-SC and BLISS-NEA

**Supplementary Figure S1.** Relative importance of contributing predictors in the entire study population. The figure describes results from a random forest variable importance analysis, with data from the pooled BLISS study population. Proteinuria was estimated by 24-hour urine protein excretion, or the urinary protein to creatinine ratio (UPCR). aCL: anticardiolipin antibody; BAFF: B cell activating factor belonging to the TNF family; BMI: body mass index; C3: complement component 3; C4: complement component 4; cSLEDAI-2K: clinical Systemic Lupus Erythematous Disease Activity Index 2000; SDI: SLICC/ACR damage index; Sm: Smith; (+): positivity.

**Cut-off for antibody positivity:**

anti-dsDNA ≥30 IU/mL

anti-Sm ≥15 U/mL

anti-ribosomal P protein >25 EU/mL

**Cut-off for low complement levels:**

C3 <90 mg/dL

C4 <16 mg/dL in BLISS-52 and BLISS-76 and <10 mg/dL in BLISS-SC and BLISS-NEA

**Supplementary Figure S2.** Relative importance of contributing predictors in patients with current or prior renal involvement (renal BILAG A−D). The figure describes results from a random forest variable importance analysis, with data from the pooled BLISS study population. Proteinuria was estimated by 24-hour urine protein excretion, or the urinary protein to creatinine ratio (UPCR). aCL: anticardiolipin antibody; BAFF: B cell activating factor belonging to the TNF family; BMI: body mass index; C3: complement component 3; C4: complement component 4; cSLEDAI-2K: clinical Systemic Lupus Erythematous Disease Activity Index 2000; SDI: SLICC/ACR damage index; Sm: Smith; (+): positivity.

**Cut-off for antibody positivity:**

anti-dsDNA ≥30 IU/mL

anti-Sm ≥15 U/mL

anti-ribosomal P protein >25 EU/mL

**Cut-off for low complement levels:**

C3 <90 mg/dL

C4 <16 mg/dL in BLISS-52 and BLISS-76 and <10 mg/dL in BLISS-SC and BLISS-NEA

**Supplementary Figure S3.** Relative importance of contributing predictors in patients with no prior renal involvement (renal BILAG E). The figure describes results from a random forest variable importance analysis, with data from the pooled BLISS study population. Proteinuria was estimated by 24-hour urine protein excretion, or the urinary protein to creatinine ratio (UPCR). aCL: anticardiolipin antibody; BAFF: B cell activating factor belonging to the TNF family; BMI: body mass index; C3: complement component 3; C4: complement component 4; cSLEDAI-2K: clinical Systemic Lupus Erythematous Disease Activity Index 2000; SDI: SLICC/ACR damage index; Sm: Smith; (+): positivity.

**Cut-off for antibody positivity:**

anti-dsDNA ≥30 IU/mL

anti-Sm ≥15 U/mL

anti-ribosomal P protein >25 EU/mL

**Cut-off for low complement levels:**

C3 <90 mg/dL

C4 <16 mg/dL in BLISS-52 and BLISS-76 and <10 mg/dL in BLISS-SC and BLISS-NEA
